# Translational and epitranscriptomic regulation of seed germination in *Arabidopsis thaliana* genotypes with contrasting dormancy phenotypes

**DOI:** 10.1007/s11103-025-01659-6

**Published:** 2025-12-04

**Authors:** J. Balarynová, B. Klčová, R. Čegan, K. Raabe, P. Krejčí, P. Bednář, D. Potěšil, V. Pustka, D. Tarkowská, V. Turečková, Z. Zdráhal, D. Honys, P. Smýkal

**Affiliations:** 1 Department of Botany, Faculty of Science, Palacký University Olomouc, Šlechtitelů 27, 779 00 Olomouc, Czech Republic; 2 Department of Plant Developmental Genetics, Institute of Biophysics, Czech Academy of Sciences, Královopolská 135, 612 00 Brno, Czech Republic; 3 Centre of Plant Structural and Functional Genomics, Institute of Experimental Botany, Czech Academy of Sciences, Šlechtitelů 31, 779 00 Olomouc, Czech Republic; 4 Laboratory of Pollen Biology, Institute of Experimental Botany, Czech Academy of Sciences, Rozvojová 263, 165 00 Prague 6, Czech Republic; 5 Department of Analytical Chemistry, Faculty of Science, Palacký University Olomouc, 17. Listopadu 12, 779 00 Olomouc, Czech Republic; 6 Mendel Centre for Plant Genomics and Proteomics, Central European Institute of Technology, Masaryk University, Kamenice 753/5, 625 00 Brno, Czech Republic; 7 Laboratory of Growth Regulators, Faculty of Science, Palacký University Olomouc and Institute of Experimental Botany, Czech Academy of Sciences, Šlechtitelů 27, 779 00 Olomouc, Czech Republic; 8 National Centre for Biomolecular Research, Faculty of Science, Masaryk University, Kamenice 753/5, 625 00 Brno, Czech Republic

**Keywords:** *Arabidopsis*, Dormancy, Germination, Polysomal profiling, Seeds, Translation

## Abstract

Seed germination represents the crucial transition from dormancy to active growth, orchestrated by environmental signals and endogenous regulatory networks. This study investigates translational regulation during germination in two *Arabidopsis thaliana* accessions with contrasting dormancy phenotypes: Columbia (Col-0), which germinates readily, and Cape Verde Islands (Cvi-0), which exhibits deep dormancy. Hormonal profiling revealed dynamic changes in abscisic acid and gibberellins, central regulators of seed dormancy. We isolated monosomal and polysomal RNA from seeds at three stages: freshly harvested (FH), after-ripened (AR), and imbibed (IM), followed by RNA sequencing. Transcriptome analysis identified ~ 14,000 mRNAs in FH seeds, increasing to 19,000 in Col and 17,000 in Cvi after imbibition. Approximately 9,000 transcripts were shared, while ~ 3,000 were genotype-specific in the monosomal fraction. Enrichment analyses indicated differential activation of translation and dormancy-related pathways. RNA modification profiling revealed N^1^-methyladenosine (m^1^A) as the most abundant mark, with higher levels in Col peaking three months post-harvest. m⁶A sequencing uncovered distinct modification landscapes, with IM seeds showing the greatest abundance and genotypic divergence in m⁶A-enriched transcripts. m⁶A peak localization correlated with genes showing differential expression between Col and Cvi. Proteomic analysis identified ~ 15,000 proteins, with components of the translational machinery enriched in IM seeds. Genotype-specific differences emerged in both monosomal and polysomal fractions. RNA-binding proteins were similar in FH and AR stages but diverged significantly upon imbibition, with Col showing enrichment in 40S ribosomal subunits, processing bodies, and RNA-associated complexes. These results reveal key translational and post-transcriptional mechanisms underpinning dormancy release and germination.

**Supplementary Information:**

The online version contains supplementary material available at 10.1007/s11103-025-01659-6.

## Key message

The study reveals translational regulation in *Arabidopsis* germination, demonstrating genotype-specific RNA modifications, gene expression, and protein enrichment, highlighting the role of m6A modification in dormancy release and germination.

**Supplementary Information:**

The online version contains supplementary material available at 10.1007/s11103-025-01659-6.

## Introduction

Angiosperm plants evolved seeds as a novel dispersal unit, sparing spores, originally used for this task in seedless plants. This improvement enabled further protection of vulnerable embryos and was accompanied by the rapid co-evolution of hidden alternation of generations, double fertilization and the establishment of completely new structures, such as flowers and seeds (Magnani 2018). Seeds are unique in terms of potential longevity, as some are reported to be alive after hundreds of years (Baskin and Baskin 2014; Sano et al*.*^2016^). Dry seeds are well equipped to survive extended periods of unfavourable conditions and contain all components required for germination and seedling establishment until the seedling reaches an autotrophic state. Mature dry seeds are dispersed in a dormant state and seed germination is triggered by hydration, i.e., the absorption of sufficient water through the seed coat in a process known as imbibition, which depends on the environmental conditions. Dormancy evolved to control the seasonal timing of seed germination (Bewley 1997). Seed germination is a highly demanding process in terms of reserve mobilization, protein synthesis and the creation of new structures. One mechanism enabling the fast and massive activation of protein synthesis is the regulation of translation at the level of mRNA storage and coordinated activation of its translation. In many model systems, including *Arabidopsis*, translation has been identified as an important regulatory checkpoint under both environmental and developmental control (Browning and Bailey-Serres 2015; Sajeev et. al. 2019). Moreover, more examples are emerging of translational regulation in response to abiotic stress in plants, such as, heat, drought, light and osmotic stress (Juntawong and Bailey-Serres 2012; Kawaguchi et al. 2004; Kage et al. 2020).

Seed-stored proteins have been studied for a long time, while seed-stored mRNAs have been studied in more detail only recently (Bai et al. 2017, 2020; Holdsworth et al. 2008a, b; Sajeev et al. 2019, Guo et al. 2023). Similarly, the degradation of a specific subset of mRNAs has for a long time been implicated in breaking seed dormancy (Dure and Waters 1965), suggesting that this process might be a prerequisite for germination (Howell et al. 2009). These stored, long-lived mRNAs have been found in many angiosperms and are believed to play crucial role in protein synthesis during germination (Basbouss-Serhal et al. 2015; Galland et al. 2014; Layat et al. 2014). A mystery about long-lived mRNAs that remains to be solved is how these are stored and protected in seeds (Sajeev et al. 2019). It has been shown that messenger ribonucleoprotein complexes (mRNPs) accumulate mRNA during seed maturation (Ajtkhozhin et al. 1976). During imbibition, monosomes containing stored mRNAs become translation-competent polysomes as revealed by polysome profiles from seeds of various plants (Marre et al*.* 1965; Spiegel et al. 1975). Recently, modifications of stored mRNAs during after-ripening have been proposed to be involved in the regulation of mRNA translation and/or degradation in the early stages of seed imbibition and subsequently program cell functioning toward germination or dormancy maintenance (Sano et al. 2020). The mechanism by which mRNAs are protected for such a long period is unknown, but it is likely due to association with RNA-binding proteins (Dedow and Bailey-Serres 2019). A crucial molecular event in dormancy loss is a change in translational machinery (Gallant et al*.* 2014; Buijs et al*.* 2019). The role of translational control has been demonstrated in *Arabidopsis* pollen tube growth (Lin et al*.* 2014) and seeds (Bai et al. 2017, 2020; Basbouss-Serhal et al. 2015). *Arabidopsis* seeds exhibit a physiological dormancy type (Finch-Savage and Leubner-Metzger 2006) based on the endogenous block regulated by plant hormone interactions, mainly the balance of abscisic acid (ABA) and gibberellins (GAs), which is usually broken by a relatively short period of cold stratification or dry seed storage and room temperature. However, large natural variation in *Arabidopsis* seed dormancy was revealed to be regulated by *DELAY OF GERMINATION 1* (*DOG-1*) locus (Alonso-Blanco et al. 2003; Bentsink et al*.* 2010) and related to environmental and geographical conditions (Vidigal et al*.* 2016). Commonly used *Arabidopsis* accessions, such as Columbia (Col) or Landsberg (Ler), are non-dormant, while others like Cape Verde Islands (Cvi) exhibit strong dormancy (Ali-Rachedi et al. 2004; Cadman et al. 2006; Finch-Savage et al. 2007). Subsequently, in natural conditions, these genotypes differ in their life strategies, ranging from germination through flowering and seed production (Martinez-Berdeja et al*.* 2020). *Arabidopsis* seeds exhibit dormancy cycling related to the environmental conditions, resulting in the establishment of a soil seed bank (Donohue 2002). Interestingly, different maternal growth conditions can result in variable dormancy phenotypes of the same genotype (Finch-Savage and Footitt 2019; Iwasaki et al. 2022) with notable differences observed in translation-related transcripts (Buijs et al*.* 2019). The most abundant modification of eukaryotic mRNA is m^6^A methylation (Shi et al. 2017), which has been suggested to play a role in plant development (Zhong et al. 2008; Růžička et al. 2017) and the regulation of gene expression (Luo et al. 2014). Moreover, m^6^A modification can influence RNA stability, decay, transport, splicing or translation (Haussmann et al. 2016; Frye et al*.* 2018; Yang et al. 2018; Luo et al. 2020). Both positive (Meyer et al*.* 2015; Wang et al*.* 2015; Shi et al. 2017) and negative (Choi et al. 2016; Qi et al. 2016) effects of m^6^A modification on RNA translation have been reported. A writer, a protein complex of the N6-adenosine methyltransferase A and B, is responsible for m^6^A modification (Reichel et al. 2019). The effect of the modification is mediated by m^6^A-binding proteins called readers represented by the YT-521B homology (YTH) domain family (Wang et al. 2014). Finally, there are modification-removing proteins, erasers, such as Alpha-ketoglutarate-dependent dioxygenase (AlkB) family proteins (Zaccara et al. 2019). The position of m^6^A modification is not random. The sites around start and stop codons, as well as 3’UTRs are the most preferred in mammalian cells (Dominissini et al. 2012; Meyer et al. 2012). The significant m^6^A modification enrichment in the 3’UTR, at stop, start codons, and a lower amount in the CDS was shown in *Arabidopsis* (Schwartz et al. 2013; Luo et al. 2014; Wang et al*.* 2015; Luo et al. 2020). However, the m^6^A modification has so far not been studied in relation to seed dormancy and germination.

The aim of this study was to elucidate the translational and post-transcriptional regulatory mechanisms governing seed dormancy release and germination in *Arabidopsis thaliana* seeds exhibiting contrasting dormancy levels: Columbia (Col-0), characterized by low dormancy, and Cape Verde Islands (Cvi-0), which exhibits deep dormancy. By integrating transcriptomic, translatomic, epitranscriptomic, and proteomic analyses across key developmental stages—freshly harvested, after-ripened, and imbibed seeds—the study sought to uncover genotype-specific differences in mRNA translation, RNA modifications, and associated protein machinery.

## Material and methods

### Plant material

Plants of *Arabidopsis thaliana* Columbia-0 (Col) and Cape Verde Islands (Cvi, genotype number N8580) were grown in 4 × 4 cm pots irrigated with tap water and fertilized weekly with standard nutrient solution (Kristalon, AGRO CS, Ltd., Czech Republic) in a growth chamber at 22/18 °C (day/night) under a 16-h day/8-h night photoperiod of artificial light (150 μmolm^−2^ s^−1^) and 70% relative humidity. Seeds were harvested upon maturation and sampled as dry, freshly-harvested (FH) or 3 months (100 days) after harvest, labelled as after-ripened (AR), and stored in the dark and at room temperature in paper bags. The sampling time points for collecting seeds were selected according to Bai et al. (2017). In addition, 3-month-old seeds were imbibed and germinated for 48 h (IM) on Whatman 1 filter paper in 90 mm Petri dishes at a 12/12 h light/dark regime and 23 °C. The period was chosen based on the results of Dekker et al*.* (2016). All experiments were conducted in triplicates. For germination assays, approximately 100–200 seeds were spread on wetted filter paper in 90 mm Petri dishes. Germination parameters were manually counted at 24, 48 and 72 h intervals (Supplementary File 1).

### Polysome profiling

The polysome profiling method (Mustroph et al. 2009; Mašek et al. 2011) was modified for seeds according to Bai et al. (2020), utilizing a 10 to 45% sucrose gradient. The sucrose solutions for 10 to 45% sucrose gradient were prepared according to Mustroph et al. (2009) and formed in a 13.2 mL open-top thin-wall polypropylene ultracentrifuge tube (Beckman, USA) using the Gradient Master™ 108 (BioComp Instruments, Canada) set for 10 to 45% w/v short cap sucrose gradient program and left overnight at 8 °C. Seed material, dry or imbibed, was weighted and immediately frozen in liquid nitrogen. Samples were either used right away or stored at -80 °C. A standardized equal weight of input tissue to buffer ratio was used for all biological replicates. This approach was based on profiles presented by Bai et al. (2017) to preserve the endogenous differences in polysome profiles during sample loading. Frozen samples were ground to powder using a mortar and pestle pre-cooled with liquid nitrogen. Polysome extraction buffer (400 mM Tris–HCl pH 9.0, 200 mM KCl, 25 mM EGTA pH 8.3, 36 mM MgCl_2_, 5 mM DTT, 5 mM PMSF, 25 µg/mL cycloheximide, 25 µg/mL chloramphenicol, 0.8% mercaptoethanol) was added in 10:1 ratio (buffer volume: sample weight). The resulting powder was left to melt on ice and further homogenized by pushing through 21G needle (B. Braun, Germany**)** five times. The homogenate was centrifuged at 16,000 × *g* for 15 min in 2 mL Eppendorf Safe-lock tubes. The supernatant was transferred to a new tube and centrifuged again. Before 500 µL of the final supernatant was loaded on top of the gradient, 200 µL of the gradient top was removed to prevent spillover. Sample tubes were placed in a SW41 Ti Beckman rotor tube bucket and centrifuged at 190,000 × *g* (~ 39,000 rpm) at 4 °C in the Optima XPN Ultracentrifuge (Beckman, USA) for 3 h (maximum acceleration, no brakes). For sample processing, the Brandel Density Gradient Fractionation System SYN-202 Syringe pump (Brandel, MD, USA) was used together with the Foxy R1 fraction collector (Teledyne ISCO, NE, USA). Absorbance signal output was recorded (flow speed 1.5 mL/min, sensitivity 0.5, baseline 10), using either UA-6 chart recorder system (Teledyne ISCO, NE, USA) or Clarity Chromatography Software (DataApex, Czech Republic) connected to the analogue output of the UA-6 detector. Fractions were collected in 2 mL Eppendorf Safe-Lock tubes, with the collector set for 30 s per tube, equal to 750 µL per fraction. Fractions collected from the sample gradients were used for protein or RNA extraction according to the protein/RNA preparation protocol by Mustroph et al. (2009). The polysome lysate input was treated identically to get the proteome/transcriptome reference.

### RNA isolation and analysis

For RNA extraction, 2 volumes of 8 M guanidine-HCl and 3 volumes of 99.8% ethanol were added to the samples, mixed well and left to precipitate theRNA at -20 °C overnight. The precipitate was then pelleted at 21,000 × *g* for 30 min at 4 °C with one 75% ethanol wash. RNA was isolated using TRI Reagent (Sigma-Aldrich, CZ) and further purificied using the Norgen Plant/Fungi Total RNA Purification Kit (Norgen, Canada) from pooled (separately monosomal and polysomal fractions) samples and sent for RNA sequencing to Novogene Ltd., Cambridge, UK. The sequencing of the libraries was conducted on an Illumina instrument with 150 bp paired-end reads (Illumina Inc. San Diego, CA, USA). mRNA read trimming based on quality (Q30) and sequencing adaptor removal were performed using Trimmomatic 0.32 (Bolger et al. 2014). Resulting high-quality reads from each library were mapped and quantified onto the *A. thaliana* reference genome GCF_000001735.4_TAIR10.1 with Araport11 annotation using RSEM (v1.3.3; Li and Dewey 2011) and with bowtie (v-1.0.0.) with default parameters. Quantified reads were used as input for differential expression analysis using the Bioconductor DESeq2 package (version 1.44.0; Love et al. 2014). Three replicates were used for each condition (Table S1). Transcripts were considered as differentially expressed when the adjusted P value was < 0.05 and log2 fold change was >  ± 1. For the analysis of mono and polysome RNAseq data, the 90th percentile of expressed genes was used instead of differential expression analysis due to the lack of replicates for all samples. Upset plots were generated using UpSetR (Gehlenborg 2019). The intersections and Venn diagrams were produced using the online tool available at https://bioinformatics.psb.ugent.be/webtools/Venn/.

The aim of our study was to capture biologically meaningful differences in translatomes across gradient subfractions and to compare them between the Col-0 and Cvi-0 accessions. In our experiment planning, we expected that the ratios, such as the PM ratio, of each subfraction abundance could differ between stages and even between accessions. Thus, equal-RNA loading would not normalise RNA for biologically driven differences in each sample subfraction composition; our equal-tissue amount strategy was a better option for this reason and enabled the extraction of each subfraction from the same biological replicate. The RNA extracted was then normalised for similar quantity prior samples being submitted to the RNA-seq. Therefore, this approach preserves absolute differences in ribosome loading while still allowing statistical comparison to fulfil our main focus, the qualitative analysis of changes between accessions and developmental stages.

### m^6^A RNA profiling and analysis

Frozen seeds were ground to a fine powder using a mortar and pestle with liquid nitrogen. Total RNA was isolated using PureLink™ Plant RNA Reagent (Thermo Fisher Scientific, MA USA). Residual DNA was removed by DNase I (Top-Bio, Czech Republic) treatment followed by phenol/chloroform extraction. EpiQuick CUT and RUN m^6^A RNA Enrichment (MeRIP) kit (Epigentek, NY, USA) was used for m^6^A RNA profiling. As an input, we used 10,000 ng of total RNA and followed the manufacturer´s instructions with one modification. After binding beads with antibody and enzymatic digestion (step 2b), isolated RNA containing m^6^A was released using RNA Clean-Up and Concentration Micro-Elute Kit (Norgen, Canada). This procedure was used instead of the RNA-binding beads suggested in the manufacturer's protocol for the EpiQuick CUT and RUN m^6^A RNA Enrichment (MeRIP) kit. In the final step of RNA Clean-UP and Concentration Kit, RNA was eluted in 11 µl of Elution Solution A. The received fractions were submitted for RNA immunoprecipitation sequencing (RIP-seq) at Novogene Ltd., Munich, Germany (Table S1). Sequencing libraries were prepared at Novogene after sample quality check, RNA fragmentation, reverse transcription, dA-tailing, adapter ligation, and PCR amplification. Resulting sequencing libraries were sequenced on an Illumina instrument with 150 bp paired-end reads (Illumina Inc., San Diego, CA, USA).

m^6^A-modified RNA read trimming and sequencing adaptor removal were done with Trimmomatic 0.32 (Bolger et al. 2014). Trimmed reads were aligned to the *A. thaliana* reference genome similarly to mRNA reads and by STAR aligner (v2.7.7a; Dobin et al*.* 2013). m^6^A peaks were identified with the Exomepeak2 package (https://github.com/ZW-xjtlu/exomePeak2) from bam files generating by RSEM mapping. Visualization and detailed analysis were done with the R package CHIPseeker (Wang et al. 2022a, b). Subsetting and combinations of dataset with DE results were done by bedtools v2.26.0 (Quinlan and Hall 2010) and upset plots were generated with UpSetR (Gehlenborg 2019). 25% TSS and 15% TTS sequences for motif analysis were extracted from the reference genome based on the CDS annotation file. The intersections and Venn diagrams were produced using the online tool available at https://bioinformatics.psb.ugent.be/webtools/Venn/. The enrichment analysis of the m^6^A-modified RNA fractions was performed using the ShinyGO 0.81 tool (Ge et al*.* 2020). The MEME Suite 5.5.7 (Bailey et al*.* 2015) was used to identify motifs in the sequences of m^6^A-modified genes. The following MEME set-up was applied: the motif length: 6–10 nucleotides, single-strand reading only.

### UHPLC-MS nucleoside analysis

RNA nucleosides (N-6-methyladenosine, N-1-methyladenosine, 5-methylcytidine, and 8-oxoguanosine) were quantified using UHPLC-MS and the protocol was modified according to the method by Fleming et al*.* (2018). RNA was digested to nucleosides in 200 µL overnight reaction at 37 °C with 100 U µL^−1^ of S1 Nuclease (Thermo Fisher Scientific) in reaction buffer (5 × reaction buffer for S1 Nuclease). This was followed by the addition of 1 U µL^−1^ of FastAP Thermosensitive Alkaline Phosphatase (Thermo Fisher Scientific) in reaction buffer (10 × FastAP Buffer) and the reaction was incubated for 1 h at 37 °C in the dark. Enzymes were removed by microfiltration (10 kDa, Amicon Ultra, Sigma-Aldrich). UHPLC-MS was performed using a Waters (Milford, MA, USA) Acquity UHPLC system with mass spectrometry detection (Select Series Cyclic IMS, Waters). Chromatographic separations were performed on a Waters T3 column (1 mm × 100 mm, 1.7 µM) and the column was operated at 45 °C. Mobile phases were water with 0.1% formic acid (A) and methanol with 0.1% formic acid (B). The analytical gradient was: 0 min, 0.1% B; 3.0 min, 0.1% B; 22 min, 55% B; 22.01 min, 97% B; 23 min, 97% B; 23.01 min, 0.1% B. The flow rate was 0.1 mL min^−1^ and the injection volume was 5 µL. The mass spectrometer was operated in positive ionization modes with a capillary spray voltage of 3.5 kV.: The source temperature was 150 °C, and the desolvation gas temperature was 220 °C. The desolvation gas flow was 600 L h^−1^, and the cone gas flow was 200 L h^−1^. Quantification was performed using calibration curves generated with authentic standards for the studied compounds, and the parameters of the calibration curves are listed in Table S2, S3.

### Proteomic analysis

To investigate the ribosome protein complexes, high-throughput liquid chromatography-mass spectrometry LC/MS was applied to both mono-/polysomal fractions (total of 36 samples, in triplicates) obtained by sucrose gradient fractionation (Col and Cvi genotypes, FH, AR and IM stages) identically as for RNAseq analysis. For protein extraction, 2 volumes of 99.8% ethanol were added, mixed and left to precipitate proteins at 4 °C overnight. Precipitate was then pelleted at 12,000 × *g* for 30 min at 4 °C, twice washed with 80% ethanol and air dried at room temperature. The pellets were stored at -80 °C. The pellets were solubilized by hot SDT buffer (4% SDS, 0.1 M DTT, 0.1 M Tris/HCl, pH 7.6) in thermomixer (Thermo Scientific, USA). The protein mixture (ca 50 μg of total protein) was used for filter-aided sample preparation (FASP) described by Wisniewski et al*.* (2018) using 1 μg of trypsin (sequencing grade; Promega). The resulting peptides were analysed by LC–MS/MS using nanoELUTE system (Bruker Co., Germany) connected to timsTOF Pro mass spectrometer (Bruker Co., Germany). Before LC separation, tryptic digests were online concentrated and desalted using a trapping column (Acclaim PepMap 100 C18, dimensions 300 μm ID, 5 mm long, 5 μm particles, Thermo Fisher Scientific, USA). After washing the trapping column with 0.1% formic acid (FA), the peptides were eluted (flow rate 300 nL/min) from the trapping column onto an analytical column (Aurora C18, 75 μm, 250 mm, 1.6 μm, Ion Opticks, Australia) by 90 min linear gradient program (3–30% of mobile phase B; mobile phase A: 0.1% FA in water; mobile phase B: 0.1% FA in 80% ACN). The linear gradient program was followed by an intensive wash of the column with 80% of the mobile phase B. The trapping and analytical column were equilibrated before sample injection into the sample loop. The analytical column was placed inside the Column Toaster (Bruker Co., Germany). According to the manufacturer's instructions, its emitter side was installed inside the CaptiveSpray ion source (Bruker Co., Germany) with the column temperature set to 40 ºC. MS/MS data were acquired in data-independent acquisition (DIA) mode with base method m/z range of 100–1700 and 1/k0 range of 0.6–1.6 V × s × cm^−2^. Enclosed DIAparameters.txt file defined m/z 400–1000 precursor range with equal windows size of 21 Th using two steps for each PASEF scan and cycle time of 100 ms locked to 100% duty cycle. DIA data were processed in DIA-NN^2^ (version 1.8) in library-free mode against the modified cRAP database (based on https://www.thegpm.org/crap/; 111 sequences in total) and UniProtKB protein database for *Arabidopsis thaliana* (version 2021/11, number of protein sequences: 27,469). No optional, but carbamidomethylation as fixed modification and trypsin/P enzyme with 1 allowed missed cleavage and peptide length 7–30 were set during the library preparation. False discovery rate (FDR) control was set to 1% FDR. MS1 and MS2 accuracies and scan window parameters were set based on the initial test searches (median value from all samples ascertained parameter values). MBR was switched on. Protein MaxLFQ intensities reported in the DIA-NN main report file were further processed using the software container environment (https://github.com/OmicsWorkflows), version 4.6.3a.

Protein Groups (PGs) were considered confidently identified in one sample if at least two replicas identified ≥ 2 unique peptides with an FDR < 1%. Isoform-specific assignments were made only when unique isoform-distinguishing peptides were detected. In cases where peptide evidence did not distinguish among isoforms, proteins were reported as protein groups. This also applies to close paralogues such as Ribosomal Proteins that commonly share 100% protein sequence identity. Protein groups were classified into total proteins, RNA-binding (Rbome) based on Zhang et al. (2023), RNA-binding (seed Rbome based on Sajeev et al*.*^2022^), 40S and 60S ribosomal subunits (Scarpin et al. 2023), eIFs/eEFs/eRFs (Browning and Bailey-Serres 2015), stress granules (Kosmacz et al. 2019), processing bodies (Xu and Chua 2011) and PABP/ALBA/ECT (Belostotsky 2003; Náprstková et al. 2021; Flores-Téllez et al. 2023) proteins.

### Analysis of ABA, GA and its metabolites

Analysis of ABA was performed according to the method described by Turečková et al. (2009) with some modifications. Briefly, approximately 3 mg of plant tissue was extracted in 1 mL ice-cold methanol/water/acetic acid (10/89/1, v/v) containing 2 pmol of mixture of stable isotope-labeled internal standards ((−)-7´,7´,7´-^2^H_3_-phaseic acid; (−)-7´,7´,7´-^2^H_3_-dihydrophaseic acid; (−) -8´,8´,8´-^2^H_3_-neophaseic acid; ( +)-4,5,8´,8´,8´-^2^H_5_-ABAGE; (−)-5,8´,8´,8´-^2^H_4_-7´-OH-ABA (National Research Council, Canada); ( +)-3´,5´,5´,7´,7´,7´-^2^H_6_-ABA (OlChemÏm,, Czech Republic). After 1 h of shaking in the dark at 4 °C, the homogenates were centrifuged (36,670 × *g*, 10 min, 4 °C), and the pellets were then re-extracted in 0.5 mL extraction solvent for 30 min. The combined extracts were purified by solid phase extraction (SPE) using Oasis™ HLB columns (30 mg, 1 mL; Waters, USA), then evaporated to dryness *in vacuo* and analysed by an Acquity UPLC® I-class system (Waters, USA) combined with Xevo™ TQ-XS triple quadrupole mass spectrometer (both Waters, UK). The sample preparation and analysis of gibberellins (GAs) were performed according to the method described in Urbanová et al. (2013) with some modifications. Briefly, tissue samples of about 5 mg FW were ground to a fine consistency using 2.7 mm zirconium oxide beads (Next Advanced Inc., ESA) and MM 400 vibration mill at frequency of 27 Hz for 3 min (Retsch GmbHGermany) with 1 mL of ice-cold 80% acetonitrile containing 5% formic acid as extraction solution. The samples were then extracted overnight at 4 °C using a benchtop laboratory rotator Stuart SB3 (Bibby Scientific Ltd., UK) after adding internal gibberellins standards ([^2^H_2_]GA_1_, [^2^H_2_]GA_4_, [^2^H_2_]GA_9_, [^2^H_2_]GA_19_, [^2^H_2_]GA_20_, [^2^H_2_]GA_24_, [^2^H_2_]GA_29_, [^2^H_2_]GA_34_ and [^2^H_2_]GA_44_) purchased from OlChemIm, Czech Republic. The homogenates were centrifuged at 36,670 × *g* and 4 °C for 10 minCorresponding supernatants were further purified using mixed-mode MAX SPE cartridges (Waters, USA) and analysed by ultra-high performance liquid chromatography-tandem mass spectrometry (UHPLC-MS/MS; Acquity UPLC® I-class system coupled to Xevo™ TQ-XS, both Waters, USA). GAs were detected using a multiple-reaction monitoring mode where the transition of the ion [M–H]^−^ to the appropriate product ion was monitored. Masslynx 4.2 software (Waters, Milford, MA, USA) was used to analyze the data, and the standard isotope dilution method (Rittenberg and Foster 1940) was used to quantify the GAs levels.

Statistical analysis was performed using the Kruskal–Wallis test, followed by post hoc non-parametric multiple comparisons (Siegel and Castellan 1988) at a 0.05 significance level using R version 4.4.3.

## Results

### ABA declines during germination, while GA levels remain stable in both genotypes

**Fig. 1:**
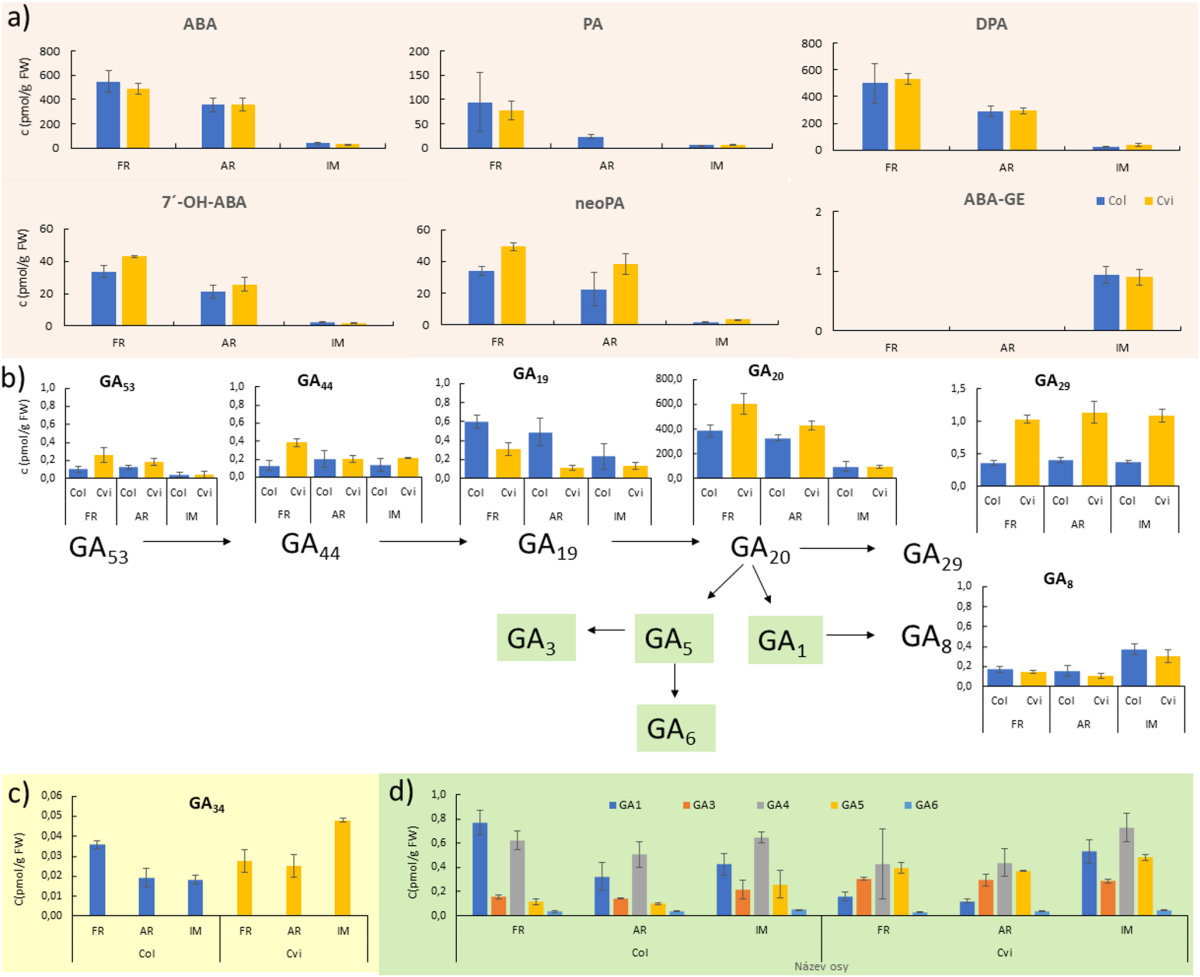
The abscisic acid (ABA) and gibberellin (GA) content in freshly harvested (FH), after-ripened (AR) and imbibed (IM) Col and Cvi seeds. **a** Quantification of ABA, phaseic acid (PA), dihydrophaseic acid (DPA), 7'-hydroxy-ABA (7´-OH-ABA), neophaseic acid (neoPA) and ABA glycosyl ester (ABA-GE). **b** The levels of GAs belonging to the 13-hydroxylation (leading to the production of GA_1_, GA_3_, GA_5_, GA_6_) gibberellin metabolic pathway, **c** GA_34_, the GA belonging to the 13-non-hydroxylated (leading to the production of GA_4_ and GA_7_) biosynthetic pathway, **d** the bioactive GAs belonging to the 13-hydroxylated (GA_1_, GA_3_, GA_5_, GA_6_) and 13-non-hydroxylated (GA_4_) pathways.. Data are expressed as median and interquartile range (three independent measurements). Different letters indicate significant differences (p = 0.05) between developmental stages of each genotype by the Kruskal–Wallis test with the following non-parametric multiple comparison test

Two *Arabidopsis* genotypes differing in seed dormancy were analysed in dry (freshly harvested FR and after-ripened AR) and 48-h imbibed (IM) stages. Columbia (Col) is non-dormant and its seeds are capable of germination immediately after shedding, while those from the Cape Verde Islands (Cvi) have deeply dormant seeds requiring after-ripening (minimum of 90–100 days, Dekkers et al. 2016) to be able to germinate. In our conditions, FH Col and Cvi seeds displayed 60% and 0% germination, respectively. However, after 3 months (90 days) after ripening, Cvi seeds germination was around 80% (Supplementary File 1). The levels of ABA and its catabolites including phaseic acid (PA), dihydrophaseic acid (DPA), 7'-hydroxy-ABA (7´-OH-ABA) and neophaseic acid (neoPA) were found to be at comparable levels in seeds of both studied *Arabidopsis* genotypes (Fig. 1a) where the lowest values were reached in IM seeds compared to FH and AR seeds. The levels of 7´-OH-ABA and neoPA, ABA catabolites formed through minor 7´-hydroxylation and 9´-hydroxylation pathways, tended to be higher in dormant Cvi seeds than non-dormant Col seeds. Another ABA catabolite, the ABA glycosyl ester (ABA-GE), was not detected in FH and AR seeds. In contrast, about 1 pmol/g FW of ABA-GE was detected in germinating seeds of both genotypes. DPA produced from PA by the main ABA catabolic 8'-hydroxylation pathway (ABA is first converted to its 8'-hydroxy form by ABA 8'-hydroxylase and then reduced in two subsequent reactions to DPA and PA by corresponding reductases) was the major ABA degradation product found in both studied *Arabidopsis* genotypes. DPA reached 300–500 pmol/g FW, which was nearly the same level as detected for ABA in dry seeds of both genotypes. In agreement with the literature, dormancy is released upon imbibition by ABA degradation; therefore, about 10 times lower ABA levels were observed in IM seeds for Col and Cvi genotypes. Notably, no significant differences were found between its level in Col and Cvi genotypes across three experimental conditions.

The levels of bioactive GAs, their inactive biosynthetic precursors and catabolites were determined as well. Among twenty profiled GAs known to be present in *Arabidopsis* (Phillips 1998), biosynthetic precursor GA_20_ was the most abundant reaching up to 600 pmol/g FW in dry seeds and about 100 pmol/g FW in imbibed seeds (Fig. 1b), which is similar situation observed for ABA (Fig. 1a). Regarding bioactive GAs, it was confirmed that GA_1_ (formed via the 13-hydroxylated pathway) and GA_4_ (the 13-non-hydroxylated pathway) are main products of GA 3-oxidase in the germinating Col and Cvi seeds (Fig. 1b, d). In Cvi seeds, however, GA_5_ (the 13-hydroxylated pathway) was also relatively abundant almost reaching GA_1_ level. The trends in the levels of GA_8_ and GA_34_ as products of degradation of bioactive GA_1_ and GA_4_ by the action of GA 2-oxidase correspond to the trends of their bioactive counterparts (Fig. 1b, c).

The RNA sequencing data were searched for the expression pattern of genes encoding enzymes of ABA and GA biosynthesis and catabolism. The genes encoding enzymes involved in GA biosynthesis (ent-kaurene synthase, GA 20-oxidase and GA 3-oxidase) were found to be expressed especially in imbibed seeds of both genotypes (Fig. S1). Besides, their expression in IM Col seeds tended to be the same or higher than in Cvi seeds, which corresponds with the lower Col seed dormancy. On the other hand, GA 2-oxidase genes (Fig. S1), responsible for the deactivation of bioactive GAs, were expressed especially in dry (FH and AR) Col seeds. GA 2-oxidase gene expression in Cvi seeds was quite stable across the studied developmental stages.

### Ribosome association and transcript profiling of mono- and polysomal fractions across seed stages differ between genotypes

**Fig. 2:**
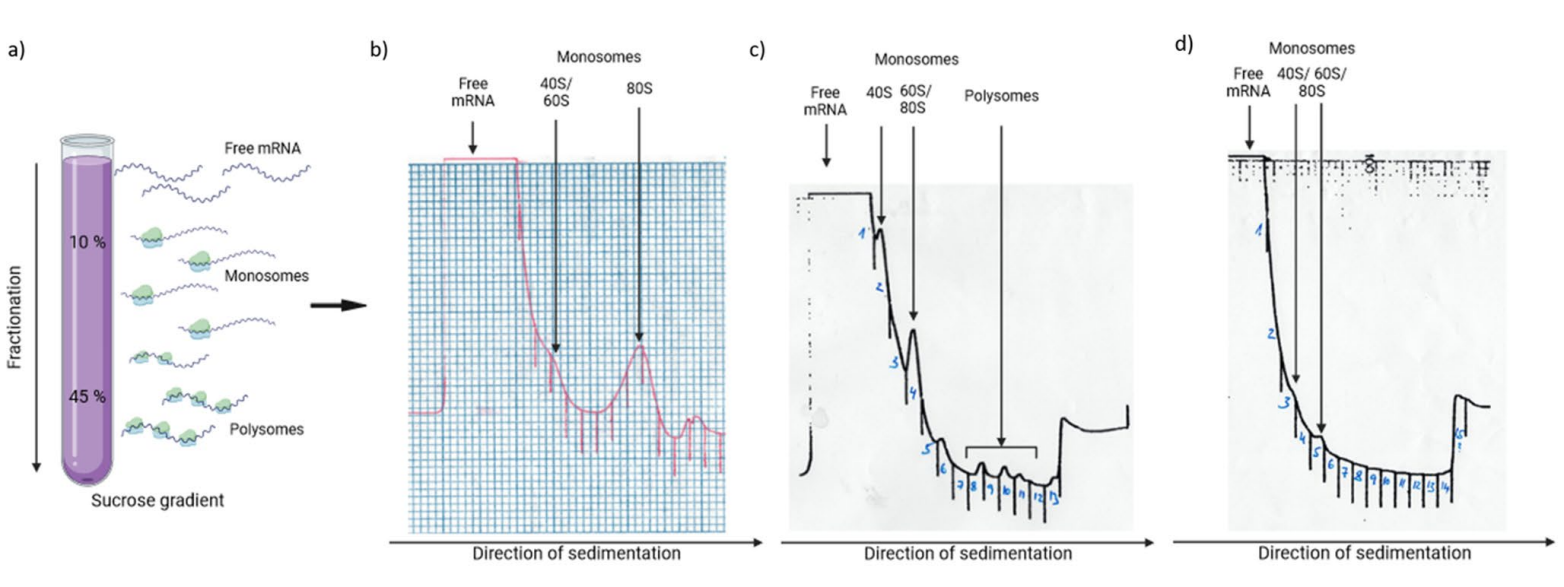
Polysome profiling. **a** The scheme of mRNA and ribosomal distribution in a 10/45% sucrose gradient used for fractionation of total seed RNA/proteins. Polysome profiles of **b** after-ripened Col seeds, **c** imbibed Col seeds, and **d** imbibed Cvi seeds. 40S/ 60S/ 80S- monosomal fractions. Marks visible on the absorbance charts represent border lines between collected fractions

**Fig. 3:**
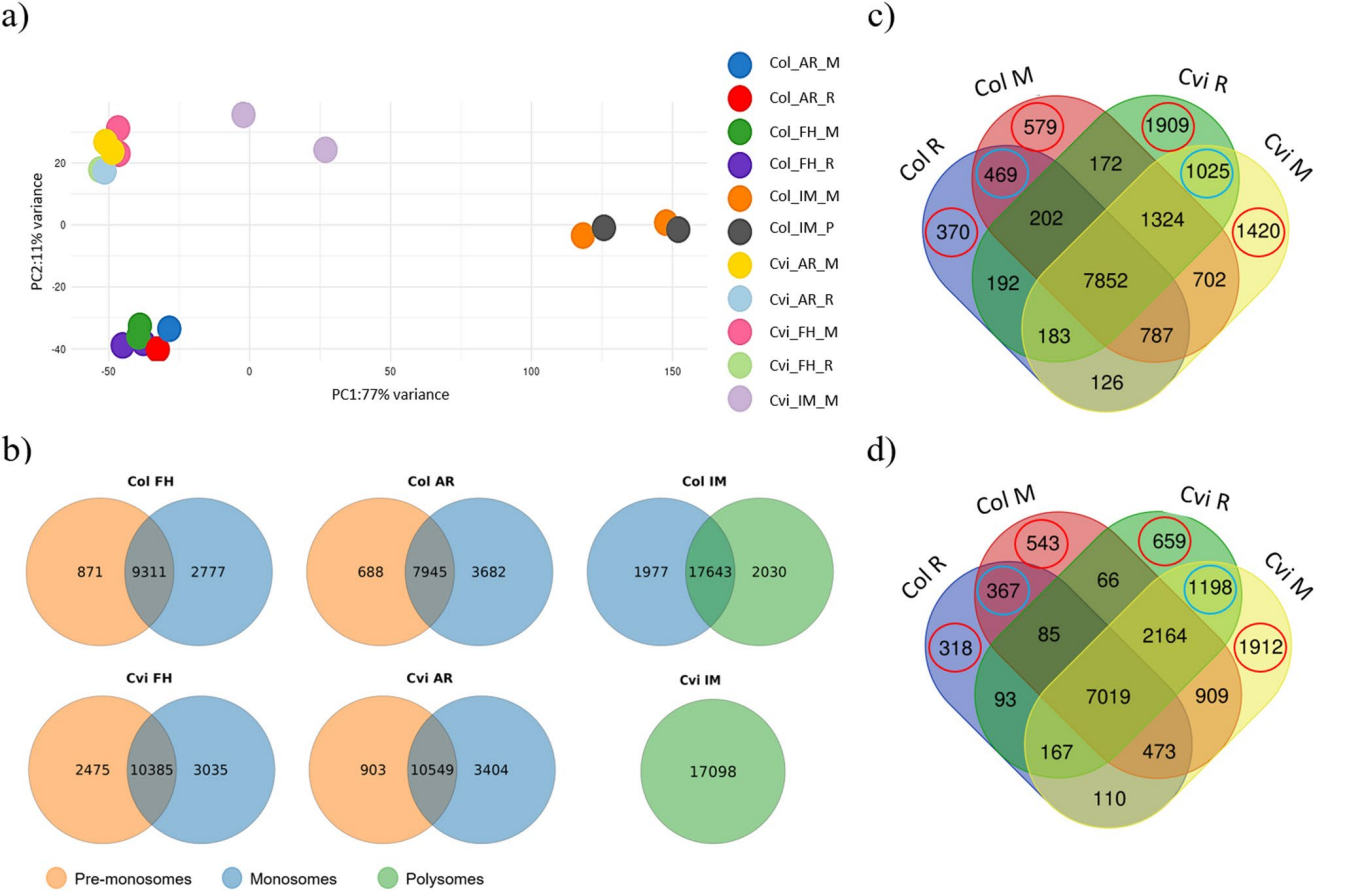
Polysome and monosome-associated RNA analysis. **a** Principal component analysis (PCA) of RNA-seq of pre-monosome (R), monosome (M), and polysome (P) associated RNA of freshly harvested (FH), after-ripened (AR) and 48-h imbibed (IM) Col and Cvi seeds. **b** Venn diagrams demonstrating specific and shared genes among fractions in Col and Cvi seeds. The upper line shows Col, and the bottom line Cvi fractions. In FH and AR seeds pre-monosomal (blue) and monosomal (red) fractions were compared. In IM seeds, monosomal (blue) and polysomal (red) fractions were analysed. **c** Venn diagrams illustrating specific and shared genes between R and M fractions of FH Col and Cvi seeds. **d** Comparison of transcripts from R and M fractions of AR Col and Cvi seeds. Red circles show genes specific for certain fractions, and blue circles genes shared between fractions of the specific genotype

To monitor and characterise ribosome association of mRNAs, we isolated mono- and polysomal fractions from freshly harvested (FH), after-ripened (3 months after harvest, AR), and 48 h imbibed (IM) Col and Cvi seeds (Fig. 2a). In FH and AR seeds of both genotypes, monosomal (M) fractions were detected, while polysomal (P) peaks (referred to two or more ribosomes bound to mRNA) were not present. In these samples, the fraction preceding monosomal peak was clearly visible and this was referred to as pre-monosomal peak (R), presumably containing lighter RNA–protein complexes such as 30S, 40S, 50S, 60S subunits of eukaryotic pre-initiation complexes (43S a 48S). Polysome profiles of these four samples (Col FH, Col AR, Cvi FH, and Cvi AR) were similar, and the representative profile is shown in Fig. 2b. The profile of 48-h imbibed Col seeds contained both M and P peaks (Fig. 2c). On the contrary, in imbibed Cvi seeds, the P peak was not detected (Fig. 2d). RNA extracted from individual fractionations was subjected to RNA-seq analysis (Table S1). The number of identified genes in Col samples ranged from 11,626 (AR) to 12,087 (FH), while in Cvi was slightly higher (13,419 in FH, and 13,952 in AR). Fractions from imbibed seeds contained 19,674 and 19,620 (Col-IM) and 17,097 (Cvi-IM) genes (Fig. S2), respectively. Although using of the Col reference for mapping Cvi reads may lead to underestimation of expression levels for some Cvi genes, we did not observe significant differences in the number of expressed genes between the two accessions (Supplementary File 2). PCA analysis showed the specific distribution of individual fractions (Fig. 3a) where FH and AR samples clustered together, while Cvi and Col genotypes were separated. Col IM stages were distinct from FH and AR samples, indicating the germination switch.

Firstly, we were interested in comparing of the respective fractions of two genotypes at different stages. In FH samples, 9,310 and 10,384 genes shared between R and M fractions were identified in Col and Cvi samples, respectively. In contrast 2,777 and 3,035 were specific to M (Fig. 3b). Similarly, in AR samples, there were 7,944 and 10,548 shared and 3,682 and 3,404 M-specific genes in Col and Cvi samples (Fig. 3b). At the IM stage, the number of genes bound to fractions rose significantly, probably pointing to a higher translation and metabolic activity. Quantitative differences are difficult to calculate because the collected fractions and respective sequencing represent different amounts of total RNA. We note that our analysis incorporates normalized expression levels, which provide a semi-quantitative estimate of transcript enrichment. Enrichment analysis was used to investigate Molecular Functions, KEGG Pathways, and Biological Processes of identified genes. Most genes isolated from the fractions were categorized as genes having nucleic acid binding, cation, and metal ion binding activity. These results suggest a correlation between polysome-binding mRNA and transcription regulation. According to KEGG pathway enrichment analysis, genes shared between fractions with no emphasis on genotype or stage are involved in metabolic pathways, biosynthesis of secondary metabolites, and ribosome function (Supplementary File 3). The fact that they are found in mono- or polysomes suggests high translation of these transcripts, therefore a demand for their functional protein products. This highlights the need for such processes during germination.

To identify shared and genotype-specific genes at different developmental stages, we compared the R and M fractions between Col and Cvi genotypes at the FH (Fig. 3c) and AR stages (Fig. 3d). In FH seeds, both genotypes shared 7,852 genes between R and M fractions (Supplementary File 4). Mentioned genes were associated with the metabolism pathway, the biosynthesis of secondary metabolites, the ribosome pathway, carbon metabolism, and with the biosynthesis of amino acids pathway. In terms of molecular function, the identified genes were categorized as follows: nucleic acid binding, cation binding, metal ion binding, RNA binding, and mRNA binding. Genes identified in fractions (R and M) and shared between both genotypes are involved in protein metabolism, gene expression regulation, and response to abiotic stress (Supplementary File 4). A total of 469 genes were shared between the R and M fractions in Col FH seeds (Supplementary File 4). In contrast, the corresponding fractions in Cvi seeds contained 1,025 genes, including six genes associated with the glycosylphosphatidylinositol (GPI)-anchor biosynthesis pathway. The molecular function categories with the highest number of genes in Cvi included nucleic acid binding, transferase activity, and cation binding. Genes found in R and M fractions of the Cvi genotype at FH stage seem to be involved in the metabolism of nucleobase-containing compounds, nucleic acids, and proteins (Supplementary File 4).

The M fraction of Col FH seeds contained unique genes categorized under RNA polymerase binding and starch binding (Supplementary File 4). In contrast, the M fraction of Cvi FH seeds had a significantly higher number of genes (1,420) than that of Col (Fig. 3c). These were mainly involved in homologous recombination, and glycan degradation (Supplementary File 4). GO term classification showed enrichment in nucleic acid binding, cation binding, and metal ion binding (Supplementary File 4). Monosome-bound genes found in Cvi FH showed an involvement in metabolism of nucleobase-containing compounds and nucleic acids, and developmental processes.

A total of 7,019 genes were shared between Col and Cvi genotypes in both R and M fractions at AR stage, classified into metabolic pathways, biosynthesis of secondary metabolites, and ribosome-related genes (Fig. 3d). Molecular functions with the highest gene numbers included nucleic acid binding, cation binding, and metal ion binding. These particular genes are potentially responsible for regulation of gene expression, protein metabolism, and cellular protein metabolism (Supplementary File 4). In AR Col seeds, M fraction genes were associated with rRNA binding, oxidoreductase activity, and NADH dehydrogenase activity. These genes were mostly involved in gene expression, macromolecule biosynthesis, and cellular amide metabolism (Supplementary File 4). The R and M fractions of Cvi AR seeds contained genes related to the mRNA surveillance pathway (Supplementary File 4). Molecular function terms included small molecule binding, nucleotide binding, and nucleoside phosphate binding. Biological functions of genes in AR Col seeds were associated with protein metabolism, cellular protein metabolism, and macromolecule modification. Unique genes in the R fraction of Cvi AR seeds were linked to cellular protein modification, protein modification, and negative regulation of biological processes (Supplementary File 4). The M fraction of Cvi AR seeds contained genes related to nucleic acid binding, transferase activity, and cation binding (Supplementary File 4). Key biological functions included nucleobase-containing compound metabolism, nucleic acid metabolism, and gene expression. Since we compared monosome/polysome association of RNA between two accessions differing in the level of seed dormancy, which substantially influences the initiation of germination in Arabidopsis seeds, the RNA-seq data were searched for expression of *DOG1* and *DOG1-like* genes (Fig. S3). The expression levels of *DOG1* were high in FH Col seeds compared to the Cvi of the same stage. However, from the individual fraction point of view, the *DOG1* expression was more pronounced in AR and IM Cvi seeds, especially in AR seeds. On the other hand, *DOG18/RDO5* gene expression was consistently high in Cvi seeds, particularly in those of the FH and AR stages. *DOG1-like* genes were mostly expressed more in FH and AR Col seeds than in those of Cvi, while in the IM seeds their levels were higher in Cvi than in Col seeds.

### Dynamics of RNA modifications and m^6^A methylation during seed storage and germination

**Fig. 4:**
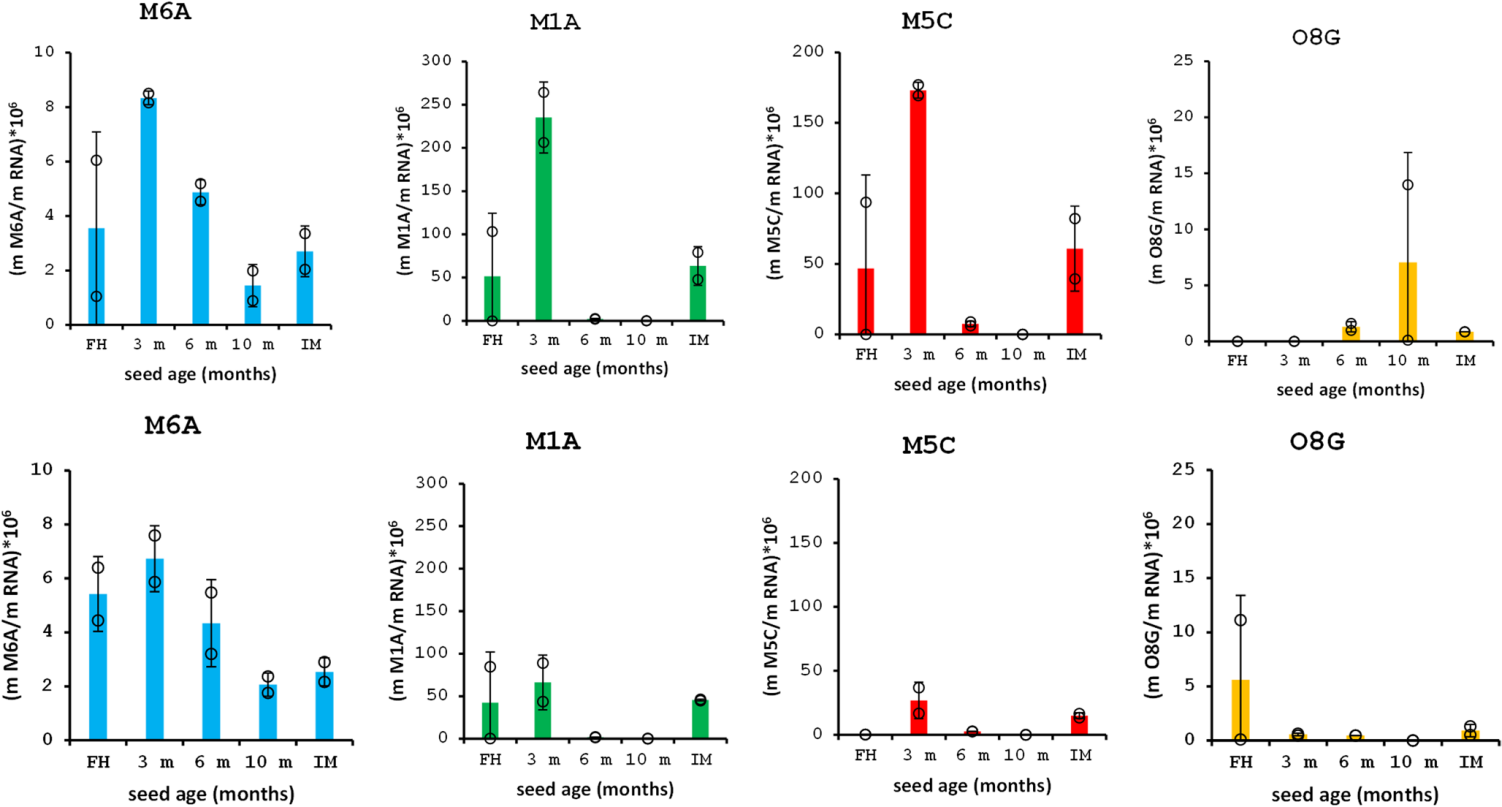
Content of m^6^A (N-6-methyladenosine), m^1^A (N-1-methyladenosine), m^5^C (5-methylcytidine) and o^8^G (8-oxoguanosine) RNA modifications in the a) Col and b) Cvi seeds just after harvesting (FH freshly harvested), after 3, 6 and 10-month ripening and in 48-h imbibed seed (after 3-month after-ripening). Data are expressed as a relation to total RNA

The RNA modifications were analysed in dry and imbibed Col and Cvi seeds. The identity of N-6-methyladenosine (m^6^A), N-1-methyladenosine (m^1^A), 5-methylcytidine (m^5^C), and 8-oxoguanosine (o^8^G) in RNA samples was confirmed by UHPLC-MS analysis (Table S2, S3). The predominant modification in dry Col seeds was m^1^A peaking at 3-month AR seeds with its content being 1.3 × a 28.2 × higher compared to m^5^C and m^5^A, respectively. The rise of o^8^G during 10 months is delayed, and its content is lower overall compared to the studied methylated bases. The dynamics of methylated base formation in Cvi seeds (i.e. a clear peak at 3 m followed by a decline in 6 m and 10 m) is similar to that observed in Col seeds, although the contents of individual bases in both seed types differ. The presence of o^8^G was also detected in Cvi seeds, although the content is, similarly to Col seeds, low and large variance among biological replicates was observed. Changes in quantities of methyl and oxidized nucleoside derivatives confirm the assumption of gradual RNA degradation during seed ageing. When respective 3-month-old seeds were left to imbibe for 48 h, the quantities of m^6^A, m^1^A, and m^5^C dropped roughly to half to a third of the original amounts in both Col and Cvi seeds (Fig. 4).

The effect of imbibition on the postharvest seed ageing was studied as well in an independent set of experiments. All four modified bases were found in imbibed seeds. Overall, the content of methylated bases in imbibed seeds grows more slowly than in their dried counterparts. The content of o^8^G in imbibed seeds is significantly lower than methylated bases, and the maximum content is reached in 6 months (Fig. S4).

**Fig. 5:**
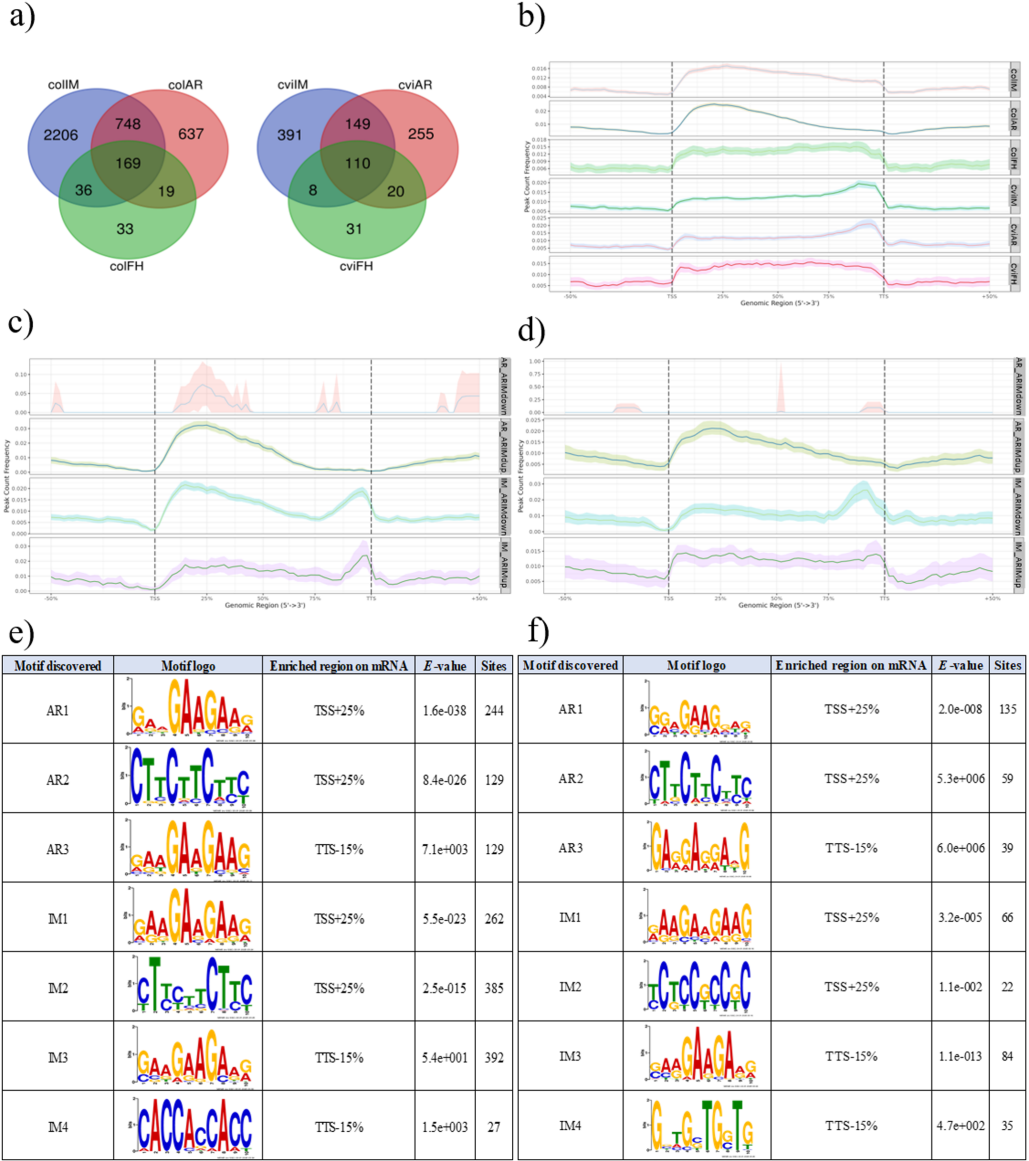
A characterization of the m^6^A methylome of Col and Cvi seeds. **a** The Venn diagrams illustrating shared and unique m^6^A-modified genes in dry (freshly harvested FH and after-ripened AR) and imbibed (IM) Col (left) and Cvi (right) seeds. **b** The position of m^6^A modification in the sequences of genes isolated from FH, AR, and IM Col and Cvi seeds. **c** The position of m^6^A modification in the sequences of Col m^6^A-modified genes. **d** The position of m^6^A modification in the sequences of Cvi m^6^A-modified genes. **e** Motifs identified in m^6^A-modified transcripts upregulated in Col seeds at AR and IM stages. **f** Motifs identified in m^6^A-modified transcripts upregulated in Cvi seeds at AR and IM stages. The enriched region was derived from the peak localization: TSS + 25%—transcription start site + 25% of the sequence length forward, TTS-15%—transcription terminator site + 15% of the sequence length backward. Significant levels are indicated by the *E*-value. Motifs were searched in MEME-suite 5.5.7 software. AR_ARIMdown- transcripts isolated from the AR stage with higher expression at the AR stage compared to the IM stage, AR_ARIMup- transcripts isolated from the AR stage with lower expression at the AR stage compared to the IM stage, IM_ARIMdown- transcripts isolated from the IM stage with higher expression at the IM stage compared to the AR stage, IM_ARIMup- transcripts isolated from the IM stage with lower expression at the IM stage compared to the AR stage

Since the m^6^A is the most prevalent modification in eukaryotic mRNA, affecting numerous aspects of mRNA metabolism (including translation), we used m^6^A sequencing to map this modification in FH as well as AR and IM Col and Cvi seeds. We used an antibody-based approach to enrich specifically with m^6^A-modified transcripts. The obtained RNA fraction was then subjected to RNAseq analysis and subsequently bioinformatically processed. First of all, the m^6^A-modified genes were compared among the studied germination stages of each genotype separately (Fig. 5a, Supplementary File 5). Afterwards, genes specific to the respective germination stage were compared between Col and Cvi seed fractions. In Col samples, the highest number of m^6^A-modified genes was detected in IM seeds, while the lowest abundance was observed in dry, FH seeds. A similar trend was found in Cvi seeds (Fig. 5a). Only two m^6^A-modified genes were shared between Col and Cvi FH seeds (Supplementary File 6). One belongs to the GRAS transcription factor family, while the other is a transposable element gene. Genes unique to Col FH seeds were associated with GO cellular component terms such as protein-containing complex and cytosol (Supplementary File 6). In contrast, genes specific to Cvi FH seeds were enriched in GO terms related to autophagy, fructose and mannose metabolism, glycolysis/gluconeogenesis, and catabolic processes (Supplementary File 6). Genes shared between Col and Cvi AR seeds (Supplementary File 6) were linked to seed oilbody biogenesis and galactolipid metabolism. Col-specific AR genes were enriched in GO terms related to metabolic pathways, biosynthesis of secondary metabolites, and response to environmental stimuli such as stress and chemicals (Supplementary File 6). In contrast, genes unique to Cvi AR seeds were associated with spliceosome and peroxisome, various catabolic processes, and reproductive development-related GO terms (Supplementary File 6). In IM seeds, Col and Cvi shared genes associated with ribosome, spliceosome, gene expression, and protein metabolic processes (Supplementary File 6). Col-specific imbibed seed genes were linked to metabolic pathways, biosynthesis of secondary metabolites, ribosome, cellular component organization and biogenesis, and localization processes (Supplementary File 6). Meanwhile, Cvi-specific imbibed seed genes were enriched in GO terms related to ribosome, carbon metabolism, spliceosome, protein metabolism, and gene expression (Supplementary File 6).

### The position of m^6^A RNA modification varies between Col and Cvi genotypes

We investigated the localization of m^6^A modification in m^6^A-modified genes and observed significant differences between Col and Cvi genotypes (Fig. 5b). Given that these genotypes differ in seed dormancy levels, we focused on comparing translationally inactive, AR seeds, and translationally active, IM seeds. In Col samples, both AR and IM seeds exhibited the highest m^6^A peaks near the start codon. In contrast, the respective stages of Cvi seeds showed m^6^A modifications clustered near the stop codon (Fig. 5b). Moreover, in the Col genotype, the frequency of the m^6^A peak is much lower at AR than at IM stage. On the other hand, in the Cvi, there is a slightly higher frequency of the m^6^A modifications at the AR in comparison to the IM stage (Fig. 5b). Finally, the frequency of m^6^A modification was found to be higher in the Cvi genotype compared to the Col. We analyzed the overall distribution of m^6^A modification throughout the sequence of all isolated transcripts and calculated the percentage of m^6^A modifications related to the position (Fig. S5). In the Col, the most prevalent position of m^6^A modification was identified within the CDS at all stages, with the highest peak at the AR stage. On the other hand, in the Cvi, the level of m^6^A modifications located within the CDS decreased at the AR and IM stages. However, the presence of m^6^A at 3'UTR rose at AR and IM stages (Fig. S5).

To explore the relationship between m^6^A peak positioning and gene expression, we analyzed the up- and downregulated m^6^A-modified genes between AR and IM seeds for each genotype (Fig. 5c, d). In Col samples, m^6^A peaks were most prominent around the transcription start site (TSS), with 25% of the sequence length showing peaks at TSS + 25 in case of transcripts isolated from the AR stage with higher expression at the AR stage compared to the expression levels of genes isolated from the IM stage (AR_ARIMup, Fig. 5c). Moreover, transcripts of IM_ARIMdown showed the highest frequency of m^6^A modification at both TSS + 25 and approximately 15% of the sequence length before the transcription termination site (TTS-15). Interestingly, transcripts of the IM_ARIMup had the peak of m^6^A modifications located mainly at the TTS + 15 site (Fig. 5c). In the Cvi genotype, AR_ARIMup transcripts contained m^6^A modification at the TSS + 25% site (Fig. 5d). In addition, the m^6^A modification peak was localized at the TTS-15% site in IM_ARIMdown transcripts. The distribution of m^6^A modification in IM_ARIMup transcripts was even (Fig. 5d). It is worth mentioning that the frequency of the m^6^A modification showed to be the highest in the case of Col AR_ARIMup and IM_ARIMup, and IM_ARIMdown transcripts, respectively (Fig. 5c, d).

The motif analysis at the TSS + 25 site within transcripts upregulated in Col AR seeds (446) indicated that GAAGAAGAAG and CTTCTTCTTC motifs were presented in 244 (55%) and 129 (29%) sequences, respectively (Fig. 5e). At TTS-15, the GAAGAAGAAG and TCTTCTTC motifs were identified in 129 and 40 sequences, respectively. According to GO terms, genes with these specific motifs at the TSS + 25 site were involved in response to stress, cellular nitrogen compound biosynthetic process, and response to chemical stimulus (Supplementary File 7). Moreover, they belong to the following Molecular function categories: metal ion, cation binding, and hydrolase activity. Notably, genes with the GAAGAAGAAG motif at the TTS-15 site were suggested to be involved in the seed dormancy and autophagy biological processes (Supplementary File 7). On the other hand, m^6^A peaks in Col IM seeds were located in two sites within CDS (Fig. 5c). In both sites, TSS + 25 and TTS-15, the motif GAAGAAGAAG was identified (Fig. 5e). From the identified 902 transcripts (genes), 262 (29%) contained this specific motif in the TSS + 25 site and 394 (44%) in the TTS-15 site. The second motif identified in 385 (43%) sequences of the TSS + 25 site was CTCCTTCTTC. In addition, the CACCACCACC motif was found in 27 (3%) sequences at the TTS-15.

The GO terms for genes containing the GAAGAAGAAG motif at both sites were similar (Supplementary File 7). In terms of Biological Functions, these genes were enriched in protein metabolism, cellular protein metabolism, and gene expression processes. Nucleic acid binding and RNA binding were the most prevalent categories of Molecular Functions. Additionally, both groups of genes were primarily associated with the ribosome pathway. Genes containing the CTCCTTCTTC motif were enriched in metabolic processes and biosynthesis of secondary metabolites according to KEGG Pathway Analysis (Supplementary File 7). This group also exhibited significant enrichment in molecular functions such as small molecule binding and nucleotide binding. Furthermore, these genes were suggested to be involved in the protein metabolic process. Genes with the CACCACCACC motif at the TTS-15 site were associated with cell wall organization, external encapsulating structure organization, and cell wall biogenesis processes (Supplementary File 7). The GO terms for this group included structural molecule activity, structural constituent of the cell wall, and demethylase activities. KEGG Pathways Analysis predicted these genes to be involved in oxidative phosphorylation and endocytosis.

In Cvi seeds, m^6^A-modified transcripts that were upregulated at the AR stage exhibited m^6^A peaks at the TSS + 25 site (Fig. 5d). Motif analysis of these genes (135 sequences) revealed that GGAGAAGGAG motif was present in all 135 sequences (100%), while CTTCTTCTTC motif appeared in 59 sequences (44%) (Fig. 5f). Additionally, at TTS-15 site, GAGGAGGAAG motif was identified in 39 sequences. Genes at the TSS + 25 site were enriched in oxidoreductase activity and ubiquitin-like protein transferase activity (Supplementary File 7) and were associated with Biological Processes such as response to stress, response to abiotic stimulus, and catabolic processes. Genes located at the TTS-15 site were linked to response to abiotic stimulus and proteolysis (Supplementary File 7). In Cvi IM seeds m^6^A-modified transcripts (171 sequences) were predominantly modified at the TTS-15 site, with a lower frequency at the TSS + 25 site (Fig. 5f). Both sites contained GAAGAAGAAG motif, with 66 (38%) sequences at TSS + 25 site and 86 (50%) sequences at TTS-15 site exhibiting this motif. The TCTCCGCCGC motif was found in 22 (13%) sequences at TSS + 25, while the GGTGCTGGTG motif appeared in 35 (20%) sequences at TTS-15. These motifs were linked to genes involved in gene expression, translation, and ribosome pathways (Supplementary File 7). In contrast, Col IM seeds showed a higher number of m^6^A-modified transcripts near the beginning of the CDS, with upregulation observed in the IM stage (Fig. 5c). This suggests that m^6^A modification at this position may play a role in the positive regulation of transcription.

### Protein composition and functional dynamics of monosome and polysome fractions across seed stages

**Fig. 6:**
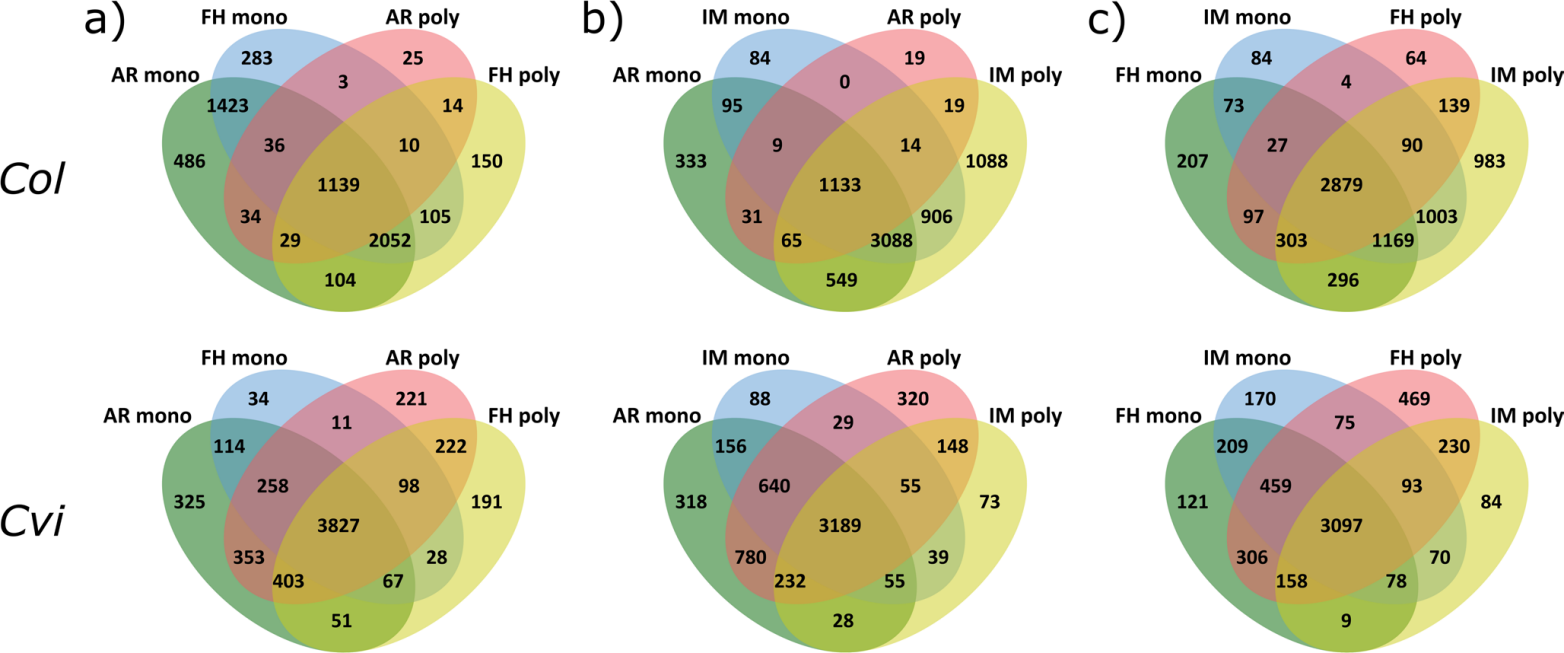
Polysome and monosome associated protein analysis. The Venn diagrams of protein comparison between monosomal and polysomal fractions of **a** freshly harvested (FH), **b** after-ripened (AR), and **c** imbibed (IM) Col and Cvi seeds

To explore proteins related to translational activation across distinct seed stages and fractions, proteins from monosome and polysome fractions were analyzed using LC–MS/MS. This analysis of translational machinery components during different stages of seed dormancy. A total of 14,488 proteins were identified. In Col seeds, monosomes contained 5,051, 5,303, and 5,329 proteins in FH, AR, and IM seeds, respectively. In Cvi seeds, monosomes contained 4,437 proteins in FH, 5,398 in AR, and 4,251 in IM. We analyzed the individual fraction proteomes by a quantitative GO enrichment (Molecular Function complete) using all identified proteins. Monosome fractions were consistently enriched in GO terms related to translation such as structural constituent of ribosome (GO:0003735) or mRNA binding (GO:0003729) among the six most enriched terms in all stages. Comparable enrichment analysis was done in polysomal fractions: Col polysomes contained 3,603 proteins in FH, AR, and IM, and Cvi polysomes contained 4,887 proteins, respectively. In Col monosomes revealed GO terms such as organic acid binding and oxidoreductase activity in FH and AR, while IM proteins were enriched in RNA helicase and ATP-dependent RNA activities. KEGG pathways across all stages included aminoacyl-tRNA biosynthesis, carbon fixation, and the TCA cycle (Supplementary File 8, Table S4, S5, S6, S7). This is likely due to certain high molecular complexes of similar size as polysomes, rather than direct association with eukaryotic translation machinery. A qualitative comparison between Col and Cvi at various stages revealed similarities in translation-related proteins but notable qualitative and quantitative differences, especially in FH and AR stages (Fig. 6a). Overall, more dynamic shifts in the shared proteome between stages, while Cvi has a more core proteome and fewer specific proteins. Specifically, the core proteome shared between AR and FH contains 1,139 proteins in Col but 3,827 in Cvi. The other notable difference between the polysomes, which in Col shared many proteins with AR and FH monosomes but not with AR and FH polysomes (Fig. 6b, c), sharing 3,088 proteins with AR and IM monosome fractions absent from AR polysomes, 549 proteins exclusively with AR monosomes and 906 exclusively with IM monosomes. Analogously, IM polysomes shared 1,169 proteins with FH and IM monosomes and smaller exclusive sets with each. In Cvi, none of the analogously shared sets exceeded 100 proteins (Fig. 6b, c). This consistency in Cvi reflects the physiological state after 48 h imbibition, when Col is already transitioned from dormancy to active growth (Fig. 6).

### RNA-binding and translational regulators

We next examined RNA-binding and translation (ribosomal proteins, translation factors) (Fig. S3). We compiled from established literature. We used it to analyse the content of our fraction, highlighting differences between Col and Cvi. Comparison of both ecotypes showed high overlap of the RPS, RPL and translation factors (eIFs, eEFs, eRFs) in monosomes, particularly in the FH stage. Polysome comparison shows a vast amount of specific translation-related proteins, consistent with their complete developmental transition upon the 48 h imbibition. The 18 and 26 RPS and RPL proteins specific to Col polysomes shows the assembly of activated ribosomes and may indicate a subpopulation involved in seed metabolism activation. The state of FH Cvi and Col seeds diverges, with only Col exhibiting actively translating ribosome subsets. Lastly, we compared the fraction proteomes with published broad and seed RBome, both of which showed a high overlap with our dataset. Among these, several key families were prominent—particularly those recognizing the m^6^A modification (m^6^A readers) or binding mRNAs critical for plant development, such as ECT (Evolutionarily Conserved C-Terminal Region), ALBA (Acetylation Lowers Binding Affinity), and PAB (Poly-A Binding). ECT proteins, containing m^6^A readers (Arribas-Hernández and Brodersen 2020), and PAB families are known interacting partners (Song et al. 2023; Reichel et al. 2024). In the sporophyte, ECT2, ECT3 and ECT4 form a functional complex that regulates important transcripts (Arribas-Hernández et al. 2018). ECT2 was found in all fractions of both Cvi and Col, while ECT3, ECT4, and ECT10 were present only in Col across stages. Notably, ECT6 was recruited to both monosome and polysomes in the IM stage, while ECT1 was specific to IM polysomes. In contrast, Cvi samples contained only ECT2, ECT5, ECT8.

PAB2, PAB4 and PAB8 in all fractions of all stages in enhancing translation efficiency, PAB2 and PAB4 interact with ECT proteins to stabilize target transcripts (Song et al. 2023). Another ECT2-interacting protein, ALBA4, was present in all fractions of both Col and Cvi. ALBA5 appeared exclusively in dormant Col monosomes but was present in both fractions after activation, suggesting a role in dormant ribonucleoprotein complexes re-engaged upon rehydration. Lastly, we observed differences in Pumilio (PUF) protein. We detected AtPUM5 AtPUM23 in all monosome and polysome fractions. Similarly, important regulator of seed maturation was abundant except in AR polysomal fraction. AtPUM6 was specific for IM Col polysomes. Overall, the functional distinctions between polysome and monosome fractions highlight the active translation in polysomes and preparatory functions during seed imbibition.

## Discussion

Seed dormancy is a critical trait shaped both by natural selection and agricultural practices (Smýkal et al. 2018). Germination is pivotal for seedling establishment and subsequent plant generations. This process relies on the longevity of seed-stored mRNAs, which are translated during germination. The separation of transcription and translation during seed maturation and germination makes seeds (and similarly pollen, Hafidh and Honys 2021) unique systems for studying developmentally regulated translational switches (Sajeev et al. 2019). In dormant stages, such as seeds and pollen, gene expression is predominantly regulated post-transcriptionally. Processes like mRNA sequestration into ribonuclear complexes and translational regulation play key roles (Hafidh and Honys 2021) and this has been studied in pollen tube growth (Hafidh et al. 2018; Klodová et al. 2023; Sze et al. 2024) and seed germination (Basbouss-Serhal et al. 2015; Layat et al. 2014; Bailey-Serres et al*.* 2009). These studies revealed significant differences in the translatomes of dormant and non-dormant seeds, driven by selective recruitment of mRNAs to polysomes and suggesting that long-lived mRNAs synthesized during seed development are stored and translated only upon imbibition (Kimura and Nambara 2010). Our study compared two *Arabidopsis* accessions representing well-established, naturally occurring extremes of seed dormancy behaviour: Col-0 typically exhibits low primary dormancy and rapid germination, while Cvi is strongly dormant and requires extensive after-ripening to germinate. This phenotypic contrast enables the identification of genotype-specific and genotype-independent transcriptional responses during dormancy release. Our primary goal was to gain insights into the genetic and transcriptional bases of dormancy variation by leveraging the natural diversity between Col-0 and Cvi. Rather than focusing solely on the effect of after-ripening within a single genotype, we aimed to dissect how dormancy-related gene expression programs differ both between and within genotypes across physiological states. This comparative approach is consistent with previous work using natural accessions to understand the genetics of complex traits such as dormancy (e.g., Bentsink et al. 2006; Kerdaffrec et al*.* 2016). We analyzed polysomal and monosomal mRNA loading at three key developmental stages: mature dry seeds (FH), after-ripened (AR) seeds, and seeds 48 h post-imbibition (IM). This work provides a comprehensive RNA-seq analysis, extending previous microarray-based studies (Basbouss-Serhal et al. 2015; Layat et al. 2014; Bai et al. 2017, 2020). Unlike earlier studies focused on a single genotype, our comparison of contrasting dormancy phenotypes highlights the functional differences in translational regulation. In agreement with previous findings (Cadman et al. 2006; Finch-Savage et al. 2007), Col seeds exhibited a germination rate of ~ 90% immediately after shedding, while Cvi seeds required an after-ripening period of ~ 100 days to achieve 65% germination (Supplementary File 1. We selected a 48-h imbibition period for analysis, stage when Col seeds had fully germinated, with root emergence and cotyledon exposure, marking a developmental transition analogous to pollen tube growth (Hafidh and Honys 2021). This stage aligns with the germination translational shift reported by Bai et al. (2017), coinciding with the full activation of the seedling developmental program.

It is well known that ABA and GAs antagonistically regulate seed dormancy and germination (Razem et al. 2006; Weiss and Ori 2007; Vanstraelen and Benková 2012). ABA promotes dormancy by inhibiting GA biosynthesis and hydrolytic enzyme activity, while GAs stimulate germination (Ogawa et al. 2003; Okamoto et al. 2006). Our results align with previous studies, showing high ABA levels in dormant seeds, moderate decline during after-ripening, and significant reduction upon imbibition (Carrera et al. 2008). DPA, the final ABA degradation product, follows this pattern. Minor ABA degradation pathways were more active in Cvi than Col, and ABA-glucose conjugation occurred only in imbibed seeds. Germination involves metabolic shifts, with GA_4_ peaking before radicle emergence (~ 40 h post-imbibition). Under our conditions (48 h, 23 °C, 12/12 h light/dark), Cvi seeds showed a ~ 30% increase in GA_4_ post-imbibition, while Col GA_4_ levels remained stable. GA_1_ was highest in FH Col seeds but decreased with imbibition. Additionally, we detected GA_3_, GA_5_, and GA_6_, with GA_5_ increasing significantly in Col during germination. Notably, both genotypes synthesized high levels of GA_20_, a precursor to bioactive GAs. Our results correlate with gene expression patterns linked to ABA/GA metabolism. Moreover, this study is the first report describing in detail all known ABA metabolites in Cvi genotype. Previously, GA 3-oxidases, the enzyme that produce the bioactive GAs, were shown to play a role in seed germination in Cvi seeds (Cadman et al. 2006, Yazdanpanah et al*.* 2017). Under our experimental conditions (germination of Col and Cvi accession for 48 h under 12/12 h light/dark regime and 23 °C) we observed a roughly 30% increase in GA_4_ level in IM Cvi seeds compared to those of dry seeds (FH and AR) (Fig. 1d). The level of GA_1_ was about threefold and fivefold higher in IM Cvi seeds than in FH and AR, respectively. In the Col genotype, GA_4_ levels were comparable in dry and germinated seeds. The GA_1_ level was highest in FH seeds, about 2.5 times lower in AR and about twofold lower in IM seeds. In addition to GA_1_ and GA_4_, we also detected bioactive GA_3_, GA_5_, and GA_6_ in both genotypes, which have not been investigated in previous studies. We found that seeds of Col and Cvi genotypes synthesize high levels of inactive precursor GA_20_, from which the bioactive GA_5_ (and subsequently GA_3_ and GA_6_) is produced to a greater extent by GA3 -oxidase (Fig. 1b). GA_5_ levels then increase significantly in the FH → AR → IM direction in the Col genotype. Although Preston et al. (2009) investigated imbibition by transcriptomic and hormone profiling using Cvi and Col genotypes, they studied only GA_4_. After seed imbibition of both genotypes, there was higher ABA content, while GA_4_ content was lower in imbibed seeds of Cvi compared with Col. Under our experimental conditions of 48 h imbibition, GA_4_ levels were approximately the same in both genotypes, as were ABA levels. Our results of the quantitative analysis of ABA, including its metabolites, and twenty GAs by UHPLC-MS/MS correlated with the expression levels of the corresponding genes encoding enzymes of appropriate biosynthetic or metabolic reactions (Fig. S1). Not only absolute ABA and GA amounts, but also the changes in the ABA/GA ratio are associated with dormancy level. The ABA/GA ratio is usually high in seeds with low germination/high dormancy. The absolute bioactive GA content shows that although Cvi seeds germination is lower than that of Col seeds, the IM Cvi seeds had a higher content of bioactive GAs. However, the proportion of various bioactive GAs differs between Col and Cvi as discussed above (Fig. 1; Table S8). Unlike absolute bioactive GA content, the ABA/GA ratios indicate the fact that dry Cvi seeds (FH and AR) are more dormant than those of Col ones (Table S9).

We identified over 10,000 genes expressed and present as mRNA in the mature seeds. This is a high proportion of the total (38,000 genes with 27,500 protein-encoding genes, TAIR10.1 genome assembly) and matches earlier studies (Basbouss-Serhal et al. 2015; Bai et al. 2017, 2020). On the other hand, there were around 5,000 proteins detected in respective monosomal and polysomal fractions. These included a large proportion of ribosomal and RNA-binding proteins. The comparison of mRNA level, translational activity, and protein abundance emphasized that selective mRNA translation is a major regulatory mechanism of seed germination (Gallard et al*.* 2014). Transcriptomic studies have documented differential accumulation of stored mRNAs during after-ripening (Basbouss-Serhal et al. 2015; Bai et al. 2017, 2020). These changes may result from transcriptional activity, mRNA turnover, or differential loading onto polysomes. In our analysis, ribosomes in dry seeds were predominantly in the monosome form, with polysomes absent. Upon 48 h imbibition, polysome peaks emerged in non-dormant Col but remained undetectable in dormant Cvi seeds, consistent with Bai et al. (2017). This supports the translational activation associated with germination in non-dormant seeds. Our focus on translatome dynamics complements earlier transcriptome studies (Buijs et al. 2020; Dekkers et al. 2013), which showed distinct gene expression profiles between seed compartments (testa, endosperm, and embryo). These compartments contribute differentially to germination, as shown by Dekkers et al. (2016), who analyzed dormant and after-ripened Cvi seeds at four time points and across seed compartments. Their work revealed early transcriptional responses in the endosperm, particularly stress-related gene categories, suggesting its protective role in dormant seeds within the soil seed bank. We also explored mRNA sequence features influencing mono/polysome distribution. U-rich motifs, particularly in the 5′ UTR, were enriched in transcripts associated with polysomes. These motifs, consistent with previous studies (Basbouss-Serhal et al. 2015; Bai et al. 2017, 2020), may facilitate the recruitment of specific RNA-binding proteins (Bai et al. 2020, 2021). Structural features such as decreased secondary structure at start and stop codons, known to enhance ribosome accessibility (Kozak 2005; Kertesz et al. 2010; Li et al*.* 2012), were also observed. A methylation of N^6^-adenosine is the most prevalent covalently bound modification of RNA (Shi et al. 2017). It is a dynamic and reversible feature possessing a wide range of regulatory functions (Meyer and Jaffrey 2014). It was proposed that m^6^A modification could be involved in the regulation of seed dormancy during after-ripening (Hu et al. 2024). In this study, we isolated mRNA from dry and imbibed Arabidopsis seeds, and using the m^6^A antibody, we obtained RNA fragments containing m6A modification. Notably, DOGL4 and DOG18 transcripts were found to carry m6A modifications exclusively in Col AR samples. DOGL4 is an abscisic acid (ABA)-induced gene that promotes the expression of specific maturation-associated genes in Arabidopsis thaliana. Although DOG1 and DOGL4 do not exhibit a direct functional relationship, their biological roles show overlap (Sall et al*.* 2019). DOG18 (RDO5) encodes a protein from the type 2C protein phosphatase family, a positive regulator of seed dormancy. This gene was identified in its mutants with low dormancy level (Xiang et al. 2014). In our study, we observed a significant reduction in the expression of both DOGL4 and DOG18 in Col seed samples during the imbibition (IM) stage (Fig. S3). By contrast, Cvi seed samples at the IM stage displayed markedly higher expression of these genes. Based on these observations, we propose that m6A modifications contribute to the post-transcriptional regulation of DOGL4 and DOG18, potentially facilitating their degradation. This mechanism may play a role in the regulation of DOG1 turnover in Arabidopsis thaliana and, consequently, in the promotion of dormancy release.

In *Arabidopsis*, higher expression of the DOG1 in freshly harvested seeds is usually associated with deeper seed dormancy, and its level was shown to decrease in after-ripened and germinating seeds (Bentsink et al*. *2010; Huo et al*.* 2016; Nakabayashi et al*.* 2012). DOG1 primarily govern seed dormancy without changing ABA and GA levels (Nakabayashi et al*.* 2012). Interestingly, we found that DOG1 was more abundant in FH Col seeds compared to those of Cvi. Since not only DOG1, but also RDO5 is proposed to be a key regulator of seed dormancy in Arabidopsis (Nakabayashi et al*.* 2012; Xiang et al. 2014), we might conclude that DOG18 (RDO5) rather than DOG1 could be the main regulator of Cvi FH dormancy acting independently of ABA and DOG1 pathways (Xiang et al*.* 2014). Besides, DOG18(RDO5) can act together (form a complex) or upstream of DOG1 in seed dormancy regulation (Yuan 2019).

It is worth mentioning the differences in levels of 8-oxo-G modifications between Col and Cvi genotypes at different stages (Fig. 4). While 8-oxo-G modifications were found to be the highest in Col samples after 10 months of after-ripening (Fig. 4a), in Cvi samples, this particular type of modification was not detected or was minimal at AR stages (Fig. 4b). Posttranscriptional modification of stored mRNA by oxidation is one of the mechanisms regulating dormancy release during the after-ripening phase as shown in sunflower seeds (Bazin et al*.* 2011). We can speculate that there is some kind of mechanism protecting mRNA from oxidation in Cvi and keeping seeds dormant. However, there is still a lack of evidence about the mechanism of dormancy release in *Arabidopsis*.

In Col IM seeds, transcripts with m^6^A peaks near the stop codon and start codon were found. On the other hand, Cvi IM seeds had the highest m^6^A peak around the stop codon and decreased peaks at other sites in comparison with Col IM seeds (Fig. 5c, d). While the overall prevalence of m^6^A modification in Col transcripts was found within the CDS, Cvi showed increased presence of m^6^A at 3'UTR during after-ripening and imbibition (Fig. S5). *Arabidopsis* mutant in m^6^A RNA demethylase (AtALKBH10B) led to the increase of m^6^A modifications around the stop codon, which negatively regulated gene expression (Wang et al. 2022a, b). Moreover, there was a decrease in m^6^A around the start codon and the rest of CDS. This finding suggested that gene expression was suppressed with a prevalence of m^6^A modifications around the stop codon. On the contrary, m^6^A modifications at a position around the start codon had a positive impact on translation in maize (Luo et al. 2020). Concerning our results obtained on Col and Cvi seeds, there is probably a connection between the position of m^6^A modification around stop and start codons with positive and negative translation regulation, respectively. We annotated transcripts with m^6^A modification and analyzed DNA motifs within coding sequences of particular genes (Fig. 5e, f; Supplementary File 5). GAA and CTT tandem repetitions occurred mostly around the start and stop codons. Zhao et al*.* (2014) revealed that in dicots, the most frequent tandem repeats within the CDS are mononucleotides A/T, dinucleotides AT, and trinucleotides AAG/CTT. There is evidence of GAA repetitive sequences found in exons in different species, such as moss (Wu et al. 2014), humans and other vertebrates (Tacke and Manley 1995), and also plants (Thomas et al. 2012). Generally, the CDS contains three-fold nucleotides tandem repeats to avoid a frame-shift mutation (Metzgar et al. 2002; Legendre et al. 2007). All of these were connected to splicing regulation.

Published proteomic studies of dormant and geminated seeds were conducted by classic 2D gel analysis, resulting in the detection of the most abundant proteins, including LEA, seed storage, heat shock, and proteins involved in energetic and protein metabolisms (Gallardo et al. 2001; Chibani et al. 2006). Our MS-based study allowed a comprehensive analysis of the entire proteome associated with monosomes or polysomes. The number of PGs identified in the Col indicates an increase in diversity of translation machinery in the polysome fraction upon imbibition. To the contrary, imbibed Cvi polysomes do not show such increase and remain similar between dormant and imbibed stages. Additionally, the GO analysis clearly shows that translational machinery is highly enriched. As expected from ribosome gradient fractionation, structural constituents of ribosomes, mRNA binding, and RNA binding proteins are among the top-enriched categories across all samples. A qualitative comparison of proteins in fractions revealed that the core shared proteome in Cvi does not follow the shift from monosomes to polysomes upon imbibition observed in Col, with Cvi maintaining a rigid protein distribution (< 100 proteins transitioning). As the focus of our analysis was eukaryotic translational machinery and its associated proteins, we primarily analyzed cytoplasmic ribosomal proteins, translation initiation factors and classes of RNA-binding proteins (RBPs) playing a pivotal role in the translational control of gene expression, acting as regulators of mRNA stability, processing, and translation in plants (Bailey-Serres 1999; Hentze et al*.* 2018; Cho et al*.* 2019; Lou et al*.* 2020; Sajeev et al. 2022). Ribosome heterogeneity adds an additional layer of complexity to translational regulation. The complexity of ribosome composition is striking; just as in *A. thaliana,* each of the 81 RPs is encoded by two to seven paralogs (Xiong et al. 2021). Our proteomic analysis revealed high complexity of 40S and 60S RPs composition that is variable between genotypes and stages. This complexity is consistent with the current understanding of ribosome heterogeneity in plants (Martinez-Seidel et al. 2020). We showed that Col has a more diverse composition of 40S and 60S subunit RPs, as well as other components of the translational machinery, compared to Cvi. This suggests that the overall higher flexibility of Col translational machinery may involve a special subpopulation of ribosomes more prone to be activated or the ability to be activated quickly. This may add to the Col FH and Col IM stages the ability to germinate within smaller time window when compared to the Cvi. The *Arabidopsis* PUMILIO (AtPUM) protein family consists of 26 members that play roles in seed development and stress responses, with functions of mediating diverse post-transcriptional processes, including ribosomal RNA processing, mRNA stability, and translation (Francischini and Quaggio 2009; Tam et al*.* 2010). Our analysis detected several PUMILIO proteins in polysomal fractions, indicating their involvement in active translation during seed germination. AtPUM9 and AtPUM11 were proposed to regulate the translation of stored mRNAs in imbibing seeds (Xiang et al*.* 2014). APUM24, which was detected in all fractions in our analysis, has been previously implicated in seed maturation and rRNA processing (Huang et al*.* 2021). Its essential role in early embryogenesis and ribosome biogenesis underscores its significance in regulating translation. Deficiencies in AtPUM24 expression lead to abnormal seed maturation and embryonic lethality (Shanmugam et al*.* 2017). These findings support our observation of AtPUM6 and AtPUM4 proteins in polysomal fractions, suggesting on their role in translation during seed dormancy and germination. In the proteomic comparison of the translational machinery between Col and Cvi, we have found differences in proteins that form m^6^A readers complexes present in both monosome and polysome fractions with some qualitative differences. This suggests that these mRNA-regulating proteins are commonly bound to the stored mRNA during the dormancy, as well as bound to the highly activated polysomal transcripts upon the activation. The m^6^A modification is recognized by proteins that direct the transcript to downstream regulation. Such proteins can be either canonical YTH-domain m^6^A readers (ECT gene family in plants) (Arribas-Hernández and Brodersen 2020), their interacting partners like ALBA family proteins or PABs family (Song et al. 2023; Reichel et al. 2024). In the sporophyte, ECTs form an interacting complex that regulates important transcripts and where the most functionally described is the ECT2/ECT3/ECT4 complex (Arribas-Hernández et al. 2018), which was further shown to directly interact with PAB2 and PAB4 or ALBA proteins (Reichel et al. 2024) binding and stabilizing m^6^A-containing mRNAs (Song et al. 2023). While the predominant ECT2 protein was found in all fractions of both Cvi and Col, ECT5 and ECT8 were the only other family members found in Cvi. Col repertoire of these regulatory proteins also included ECT4 and ECT10 unique to Col monosomes and ECT6 unique to IM polysomes and completely lacking the ECT8. The presence of various ECTs in the mRNA-binding pool of proteins in the seed developmental stages indicates the m^6^A-binding ECT complex has different composition than the well described sporophytic complex. Genes encoding ECT5, ECT8 and ECT10 are highly expressed during the pollen development (Klodová et al. 2023) which possibly links these ECT members to be present in dormant stages of plant life cycle. In our dataset, we also detected PAB2, PAB4 and PAB8 proteins. These were shown to enhance translation efficiency and to interact with the ECT proteins and maintain stability of transcripts (Song et al. 2023). Three PABs detected in our analysis are highly expressed in most of *Arabidopsis* tissues and their double and triple mutants show developmental defects and embryo lethality, respectively (Zhao et al. 2019). The presence of PABs in both dormant and activated translation machinery supports both hypotheses: i) to be mRNA stabilizing factors for mRNA maintenance in the dormant stage, ii) to enhance translation after rehydration and translation activation. ALBA-family (Acetylation lowers binding affinity) proteins belong to an ancient group of small basic proteins with the capacity to bind RNA and regulate developmental processes, including seedling development (Goyal et al. 2016; Magwanga et al. 2019; Náprstková et al. 2021; Tong et al. 2022). We detected the ALBA4 protein both in Col and Cvi genotypes and in monosome and polysome fractions. ALBA4 was shown to be interacting with ECT2. Thus, these two proteins found could be the core m^6^A-binding partners to which the more functionally specialized components like other ALBA, ECT or PABs join. ALBA5 was present only in monosomes in dormant stages (FH and AR), while it was both in monosomes and polysomes in IM stage. This suggests that the ALBA5 could be one of the factors that are part of the dormant ribonucleoparticles which are activated after seed rehydration. Our proteomic analysis establishes translational proteome reorganization capacity as a component of seed dormancy. The identification of specific regulatory proteins provides insight into seeds with similar transcriptomes and hormone profiles exhibit opposite germination phenotypes. These findings highlight the focus from gene expression to post-translational control as an important regulatory layer. Fully deciphering the molecular mechanisms that activate or restrict proteome reorganization in dormant seeds by focusing on regulatory players found in our proteomic data represents a critical next step for understanding seed dormancy and germination timing.

In conclusion, our findings provide new insights into the translational dynamics underlying seed dormancy and germination. By elucidating the interplay between mRNA storage, post-transcriptional regulation, and polysome recruitment, this study advances our understanding of the molecular mechanisms driving seedling establishment. It offers potential targets for improving seed performance in agricultural contexts.

## Supplementary Information

Below is the link to the electronic supplementary material.

Supplementary Material 1

Supplementary Material 2

Supplementary Material 3

Supplementary Material 4

Supplementary Material 5

Supplementary Material 6

Supplementary Material 7

Supplementary Material 8

Supplementary Material 9

Supplementary Material 10

Supplementary Material 11

## Data Availability

The transcriptome sequencing data are deposited in the European Nucleotide Archive (https://www.ebi.ac.uk/ena) under accession number PRJEB86102, and the mass spectrometry proteomics data have been deposited in the ProteomeXchange Consortium via the PRIDE partner repository under the PXD060515 genotype.
