# Supplementary figures and images for: Translational and epitranscriptomic regulation of seed germination in Arabidopsis thaliana genotypes with contrasting dormancy phenotypes
Source: Plant Mol Biol. 2025 Dec 4;115(6):135. doi: 10.1007/s11103-025-01659-6 (PMC12678530; doi:10.1007/s11103-025-01659-6)

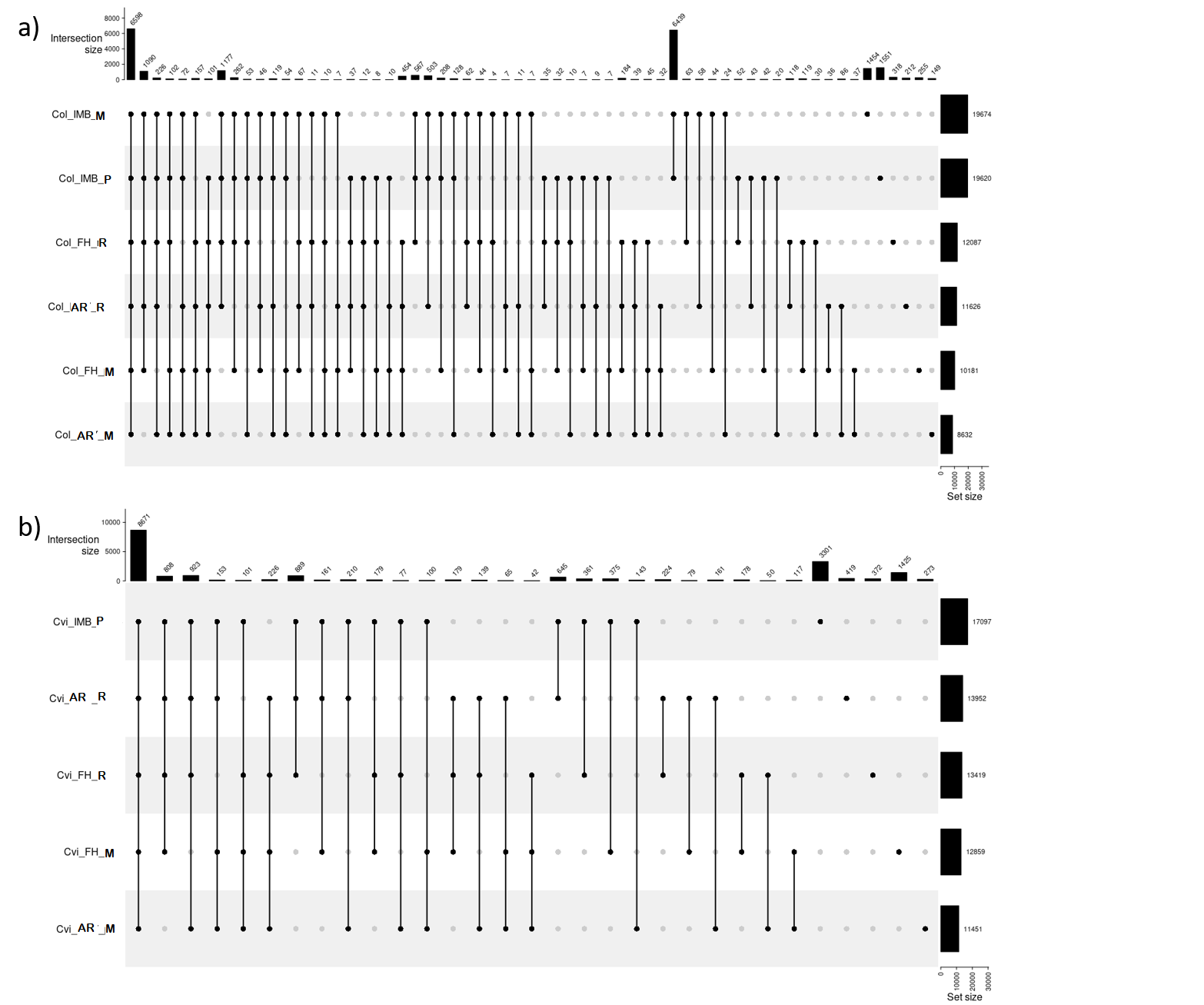

Supplement: Supplementary file 1 — Supplementary Material 1 [file 11103_2025_1659_MOESM1_ESM.tif]

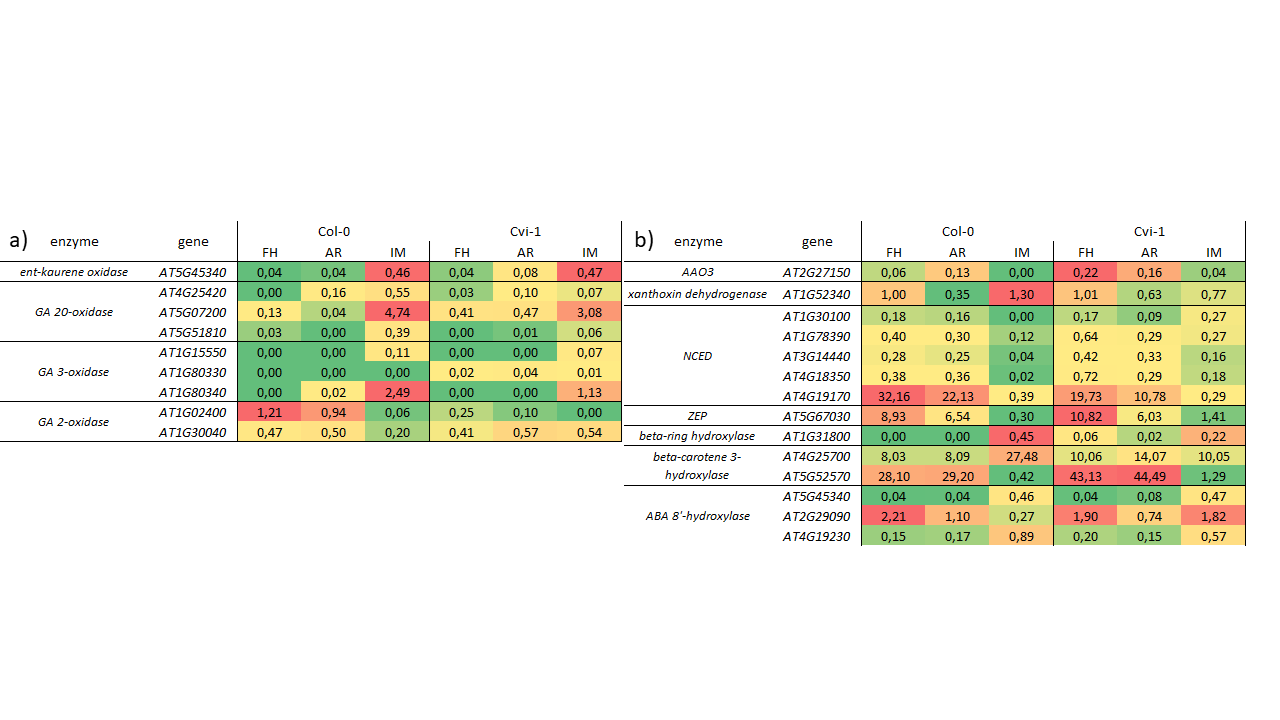

Supplement: Supplementary file 2 — Supplementary Material 2 [file 11103_2025_1659_MOESM2_ESM.tif]
