# Supplementary material for: Translational and epitranscriptomic regulation of seed germination in Arabidopsis thaliana genotypes with contrasting dormancy phenotypes
Source: Plant Mol Biol. 2025 Dec 4;115(6):135. doi: 10.1007/s11103-025-01659-6 (PMC12678530; doi:10.1007/s11103-025-01659-6)
Supplement: Supplementary file 3 — Supplementary Material 3 [file 11103_2025_1659_MOESM3_ESM.docx]

**Supplementary material**

**Translational and epitranscriptomic regulation of seed germination in *Arabidopsis thaliana* genotypes with contrasting dormancy phenotypes.**

Balarynová J.^1*^, Klčová B.^1*^, Čegan R.^2,3^, Raabe K.^4^, Krejčí P. ^5^, Bednář P. ^5^, Potěšil D. ^6^, Pustka V. ^6^, Tarkowská D.^7^, Turečková V.^7^, Zdráhal Z.^6,8^, Honys D.^4^, Smýkal P.^1^

Journal: Plant Molecular Biology

**Supplementary Tables**

**Table S1.** List of samples subjected to RNAseq and m^6^A RIPseq analysis. AR – after-ripened, FH- freshly harvested, IM- imbibed, M- monosomal fraction, P- polysomal fraction, R- pre-monosomal fraction, WS- whole seed.

**Table S2.** Characterization of calibration curves of studied modified nucleosides.

| **Nucleoside** | **Regression function** | **Reliability value (R2)** |
| --- | --- | --- |
| N-6-methyladenosine | y = 915443x + 2543.4 | 0.9997 |
| N-1-methyladenosine | y = 117821x – 24.691 | 0.9997 |
| 5-methylcytidine | y = 8128.4x + 70.061 | 0.9945 |
| 8-oxoguanosine | y = 41578x – 11.478 | 0.9997 |

**Table S3.** Selected standards of modified nucleosides and their characteristic retention times and m/z signals (in positive ionization mode).

| **Nucleoside** | **Retention time (min)** | **Signal (m/z)** |
| --- | --- | --- |
| N-6-methyladenosine | 12.74 | 282.1202 |
| N-1-methyladenosine | 7.59 | 282.1202 |
| 5-methylcytidine | 7.69 | 258.1096 |
| 8-oxoguanosine | 10.89 | 300.0948 |

**Table S4.** The comparison of the protein composition of monosomal fraction among freshly harvested, after-ripened and imbibed Col seeds and results of the enrichment analysis. FH: freshly harvested seeds, AR: after-ripened seeds; IM: imbibed seeds, 1:present, 0:absent

| **FH** | **AR** | **IM** | **number of proteins** | **enriched KEEG pathway** |
| --- | --- | --- | --- | --- |
| **1** | **0** |  | 130 | no matching pathway |
| **1** | **0** | **0** | 64 | Steroid biosynthesis |
| **1** |  | **0** | 455 | Ether lipid metabolism; Cutin suberin and wax biosynthesis; Glycerophospholipid metabolism; Phenylpropanoid biosynthesis; Protein processing in endoplasmic ruticulum; Biosynthesis of secondary metabolites |
| **0** | **1** |  | 254 | Nucleotide excision repair |
| **0** | **1** | **0** | 85 | no matching pathway |
|  | **1** | **0** | 494 | Glycosphingolipid biosynthesis; Phenylpropanoid biosynthesis; Biosynthesis of secondary metabolites |
|  | **0** | **1** | 634 | Mismatch repair; ABC transporters; Ubiquinone and other terpenoid-quinone biosynthesis; Porphyrin metabolism; Phenylpropanoid biosynthesis; Ascorbate and aldarate metabolism |
| **0** |  | **1** | 780 | DNA replication; Mismatch repair; Ascorbate and aldarate metabolism; ABC transporters; phenylpropanoid biosynthesis |
| **0** | **0** | **1** | 517 | Mismatch repair; Glucosinolate biosynthesis; Porphyrin metabolism; DNA replication; ABC transporters; Phenylpropanoid biosynthesis |
| **1** | **1** | **0** | 308 | Cutin suberine and wax biosynthesis; Phenylpropanoid biosynthesis; Biosynthesis of secondary metabolites |
| **1** | **0** | **1** | 41 | no matching pathway |
| **0** | **1** | **1** | 118 | Nucleotide excision repair |

**Table S5.** The comparison of the protein composition of **monosomal fraction** among freshly harvested, after-ripened and imbibed Cvi seeds and results of the enrichment analysis. Abbreviations: FH: freshly harvested seeds, AR: after-ripened seeds; IM: imbibed seeds, 1:present, 0:absent

| **FH** | **AR** | **IM** | **number of proteins** | **enriched KEEG pathway** |
| --- | --- | --- | --- | --- |
| **1** | **0** |  | 56 | Porphyrin metabolism; Spliceosome |
| **1** | **0** | **0** | 13 | Zeatin biosynthesis; Fatty acid degradation; Porphyrin metabolism |
| **1** |  | **0** | 291 | Non-homologous end-joining; Zeatin biosynthesis; Ubiquinone and other terpenoid-quinone biosynthesis; Arginine and proline metabolism; Fatty acid biosynthesis |
| **0** | **1** |  | 492 | Circadian rhythm-plant; Nitrogen metabolism; Phenylpropanoid biosynthesis; Biosynthesis of secondary metabolites |
| **0** | **1** | **0** | 322 | Biosynthesis of secondary metabolites |
|  | **1** | **0** | 837 | Arginine and proline metabolism; Ubiquinone and other terpenoid-quinone biosynthesis; Biosynthesis of nucleotide sugars; Amino and nucleotide sugar metabolism; Fatty acid metabolism |
|  | **0** | **1** | 95 | no matching pathway |
| **0** |  | **1** | 145 | no matching pathway |
| **0** | **0** | **1** | 43 | no matching pathway |
| **1** | **1** | **0** | 236 | Non-homologous end-joining; Ubiquinone and other terpenoid-quinone biosynthesis; Arginine and proline metabolism; Biosynthesis of nucleotide sugars; Amino sugar and nucleotide sugar metabolism |
| **1** | **0** | **1** | 27 | Spliceome |
| **0** | **1** | **1** | 76 | no matching pathway |

**Table S6.** The comparison of the protein composition of polysomal fraction among freshly harvested, after-ripened and imbibed Col seeds and results of the enrichment analysis. FH: freshly harvested seeds, AR: after-ripened seeds; IM: imbibed seeds, 1:present, 0:absent

| **FH** | **AR** | **IM** | **number of proteins** | **enriched KEEG pathway** |
| --- | --- | --- | --- | --- |
| **1** | **0** |  | 201 | Ribosome |
| **1** | **0** | **0** | 37 | Monoterpenoid biosynthesis; Brassinosteroid biosynthesis; Fatty acid biosynthesis; Ribosome |
| **1** |  | **0** | 100 | Fatty acid biosynthesis; Phenylpropanoid biosynthesis; Biosynthesis of secondary metabolites |
| **0** | **1** |  | 22 | no matching pathway |
| **0** | **1** | **0** | 2 | no matching pathway |
|  | **1** | **0** | 28 | Phenylalanine metabolism; Cutin suberine and wax biosynthesis; Phenylpropanoid biosynthesis |
|  | **0** | **1** | 1636 | Porphyrin metabolism; Sulfur metabolism; ABC transporters; Phenylpropanoid biosynthesis; Glycerophospholipid metabolism; Oxidative phosphorylation |
| **0** |  | **1** | 1652 | DNA replication; Porphyrin metabolism; Folate biosynthesis; Sulfur metabolism; Nucleotide Excision repair; Phenylpropanoid biosynthesis |
| **0** | **0** | **1** | 1167 | Porphyrin metabolism; Sulfur metabolism; DNA replication; Phenylpropanoid biosynthesis; Ascorbate and alderate metabolism; Biosynthesis of secondary metabolites |
| **1** | **1** | **0** | 18 | Phenylalanine metabolism; Phenylpropanoid biosynthesis |
| **1** | **0** | **1** | 143 | Oxidative phosphorylation; Endocytosis; Ribosome |
| **0** | **1** | **1** | 13 | no matching pathway |

**Table S7:** The comparison of the protein composition of polysomal fraction among freshly harvested, after-ripened and imbibed Cvi seeds and results of the enrichment analysis. FH: freshly harvested seeds, AR: after-ripened seeds; IM: imbibed seeds, 1:present, 0:absent

| **FH** | **AR** | **IM** | **number of proteins** | **enriched KEEG pathway** |
| --- | --- | --- | --- | --- |
| **1** | **0** |  | 96 | no matching pathway |
| **1** | **0** | **0** | 52 | no matching pathway |
| **1** |  | **0** | 602 | Thiamine metabolism; Biotin metabolism; Panthothenate and CoA biosynthesis; Arginine and proline metabolism; Photosynthesis; Inositol phosphate metabolism; Fructose and mannose metabolism |
| **0** | **1** |  | 255 | Biosynthesis of secondary metabolites |
| **0** | **1** | **0** | 194 | Nitrogen metabolism; Biosynthesis of amino acids; Biosynthesis of secondary metabolites |
|  | **1** | **0** | 984 | Non-homologous end-joining; Steroid biosynthesis; Thiamine metabolism; Biotine metabolism; Folate biosynthesis; Photosynthesis; Porphyrin metabolism; Arginine and proline metabolism |
|  | **0** | **1** | 73 | Protein processing in endoplasmic reticulum |
| **0** |  | **1** | 85 | Protein processing in endoplasmic reticulum |
| **0** | **0** | **1** | 36 | Protein processing in endoplasmic reticulum |
| **1** | **1** | **0** | 448 | Thiamine metabolism; Biotin metabolism; Arginine and proline metabolism; Various types of N-glycan biosynthesis; Pantothenate and CoA biosynthesis; Photosynthesis |
| **1** | **0** | **1** | 22 | no matching pathway |
| **0** | **1** | **1** | 28 | no matching pathway |

**Table S8.** The quantities of detected bioactive gibberellins in Col and Cvi seeds and their absolute amount (sum).

| sample/GA (pmol/g FW) | | **GA_1_** | **GA_3_** | **GA_4_** | **GA_5_** | **GA_6_** | ***sum*** |
| --- | --- | --- | --- | --- | --- | --- | --- |
| **Col** | **FH** | 0,77 | 0,16 | 0,62 | 0,11 | 0,03 | **1,70** |
|  | **AR** | 0,32 | 0,14 | 0,51 | 0,10 | 0,04 | **1,11** |
|  | **IM** | 0,42 | 0,21 | 0,65 | 0,26 | 0,05 | **1,60** |
| **Cvi** | **FH** | 0,16 | 0,31 | 0,43 | 0,40 | 0,03 | **1,32** |
|  | **AR** | 0,12 | 0,30 | 0,44 | 0,37 | 0,04 | **1,27** |
|  | **IM** | 0,53 | 0,29 | 0,73 | 0,48 | 0,04 | **2,07** |

**Table S9.** The ABA/GA ratio in Col and Cvi seeds.

| sample | | ABA (pmol/g FW) | GA (pmol/g FW) | ABA/GA |
| --- | --- | --- | --- | --- |
| **Col** | **FH** | 547,00 | 1,70 | **321,76** |
|  | **AR** | 354,63 | 1,11 | **318,74** |
|  | **IM** | 38,66 | 1,60 | **24,22** |
| **Cvi** | **FH** | 486,83 | 1,32 | **369,77** |
|  | **AR** | 358,55 | 1,27 | **282,66** |
|  | **IM** | 30,68 | 2,07 | **14,80** |

**Supplementary Figures**


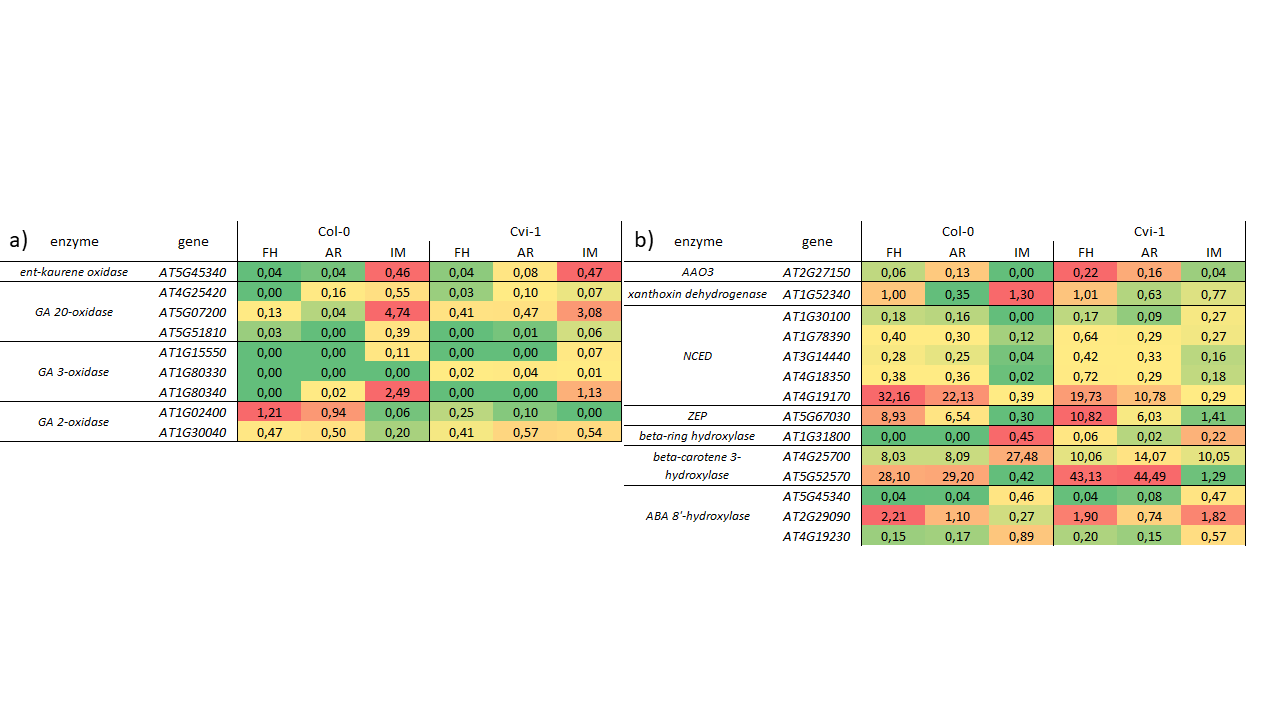


**Fig. S1.** The heatmap of genes encoding enzymes involved in biosynthesis and catabolism of ABA (**a**) and GAs (**b**) in the dry freshly harvested (FH), dry after-ripened (AR) and imbibed (IM) Col and Cvi seeds. The heatmap is based on average TMM (Trimmed Mean of M values) values from RNA sequencing. The red colours are used to represent larger values, while the green colours represent smaller values of gene expression for each group of genes encoding the same enzyme. Legend: ZEP: zeaxanthin epoxidase; NCED: 9-*cis*-epoxycarotenoid dioxygenase; SDR1: short-chain dehydrogenase reductase; AAO3: abscisic aldehyde oxidase.


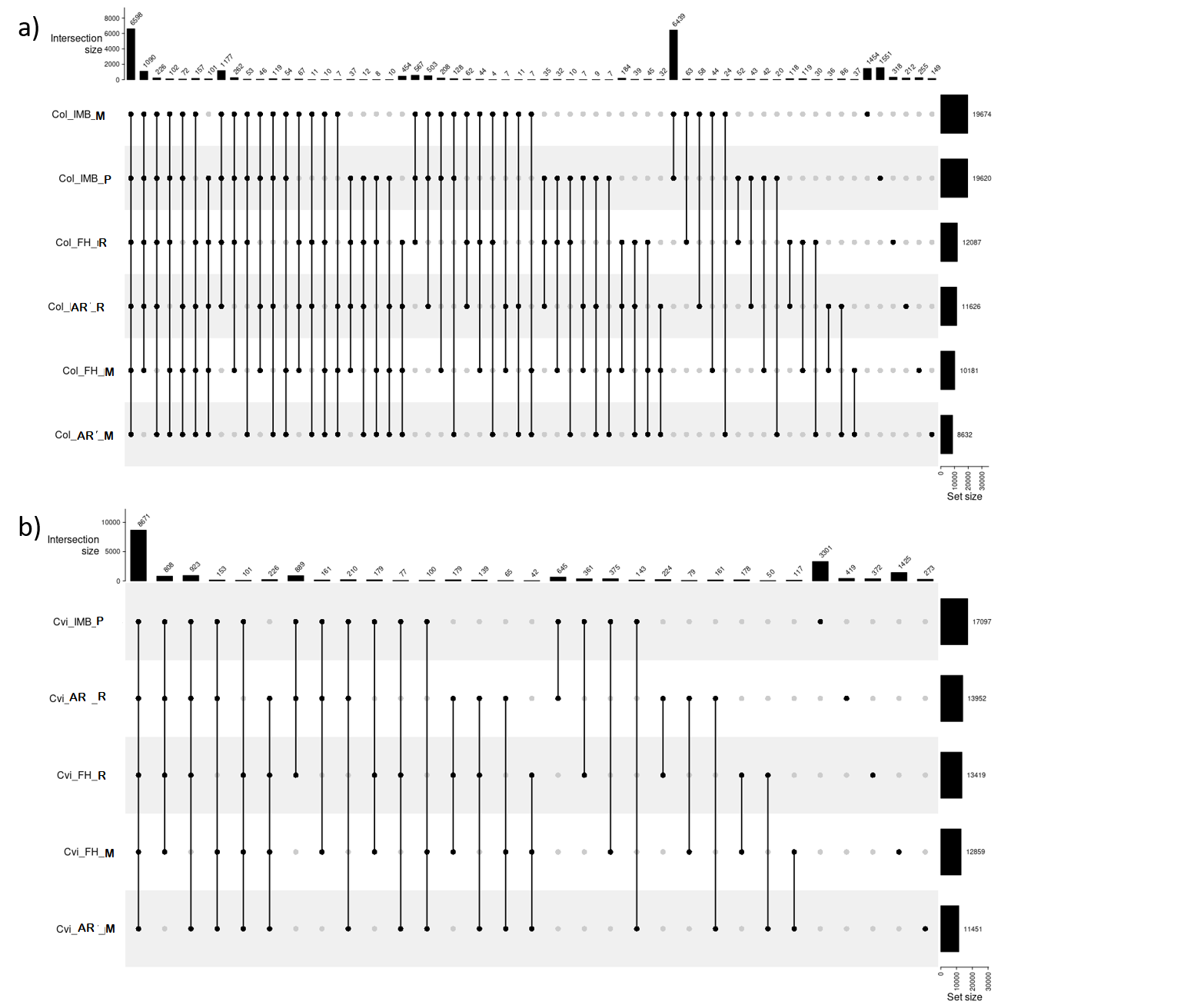


**Fig. S2.** Upset plot representing the relationship of pre-monosomal (R), monosomal (M), and polysomal (P) genes from freshly harvested (FH), after-ripened (AR), and imbibed (IM) Col (a) and Cvi (b) seeds.

A

| gene | | whole seeds | | | | | |
| --- | --- | --- | --- | --- | --- | --- | --- |
|  |  | col | | | cvi | | |
|  |  | FH | AR | IM | FH | AR | IM |
| *DOG1* | *AT5G45830* | 45,69 | 51,68 | 0,54 | 25,72 | 34,33 | 0,93 |
|  |  |  |  |  |  |  |  |
| *DOG1-LIKE 3* | *AT4G18690* | 0,25 | 1,07 | 3,13 | 1,07 | 1,22 | 25,95 |
| *DOG1-LIKE 2* | *AT4G18680* | 132,55 | 91,30 | 1,92 | 32,15 | 26,36 | 7,29 |
| *DOG1-LIKE 1* | *AT4G18660* | 5,93 | 11,38 | 0,31 | 6,05 | 2,91 | 0,27 |
| *DOG1-LIKE 4* | *AT4G18650* | 87,21 | 66,98 | 5,53 | 24,58 | 19,03 | 11,97 |
|  |  |  |  |  |  |  |  |
| *DOG18/RDO5* | *AT4G11040* | 97,60 | 58,47 | 23,44 | 185,02 | 161,83 | 82,79 |

B

| gene | | FH seeds | | | |  | AR seeds | | | |  | IM seeds | | |
| --- | --- | --- | --- | --- | --- | --- | --- | --- | --- | --- | --- | --- | --- | --- |
|  |  | col | | cvi | |  | col | | cvi | |  | col | | cvi |
|  |  | R | M | R | M |  | R | M | R | M |  | M | P | M |
| *DOG1* | *AT5G45830* | 99,82 | 147,62 | 12,85 | 25,01 |  | 29,53 | 47,76 | 133,11 | 104,00 |  | 0,05 | 0,01 | 4,96 |
|  |  |  |  |  |  |  |  |  |  |  |  |  |  |  |
| *DOG1-LIKE 3* | *AT4G18690* | 0,42 | 0,54 | 0,00 | 0,77 |  | 5,49 | 6,19 | 0,94 | 1,44 |  | 0,24 | 0,42 | 17,63 |
| *DOG1-LIKE 2* | *AT4G18680* | 92,41 | 137,36 | 43,91 | 42,14 |  | 117,28 | 161,22 | 55,87 | 48,66 |  | 0,06 | 0,06 | 44,44 |
| *DOG1-LIKE 1* | *AT4G18660* | 9,36 | 12,86 | 5,20 | 6,20 |  | 19,30 | 22,88 | 6,93 | 8,01 |  | 0,12 | 0,14 | 0,91 |
| *DOG1-LIKE 4* | *AT4G18650* | 310,58 | 242,88 | 34,11 | 47,72 |  | 218,53 | 178,26 | 51,69 | 37,85 |  | 0,72 | 1,58 | 25,68 |
|  |  |  |  |  |  |  |  |  |  |  |  |  |  |  |
| *DOG18/RDO5* | *AT4G11040* | 67,64 | 99,85 | 95,76 | 137,66 |  | 102,11 | 186,35 | 226,72 | 254,98 |  | 5,95 | 4,70 | 72,52 |

**Fig. S3.** The heatmap of genes encoding DOG1 (Delay Of Germination 1), DOG1-like and DOG18/RDO5 in the dry freshly harvested (FH), dry after-ripened (AR) and imbibed (IM) Col and Cvi seeds. Their expression is shown in both whole seeds (A) and also in fractions (B), R:pre-ribosomal fraction, M: monosomal fraction, P: polysomal fraction. The heatmap is based on average TMM (Trimmed Mean of M values) values from RNA sequencing. The red colours are used to represent larger values, while the green colours represent smaller values of gene expression for each group of genes encoding the same enzyme.


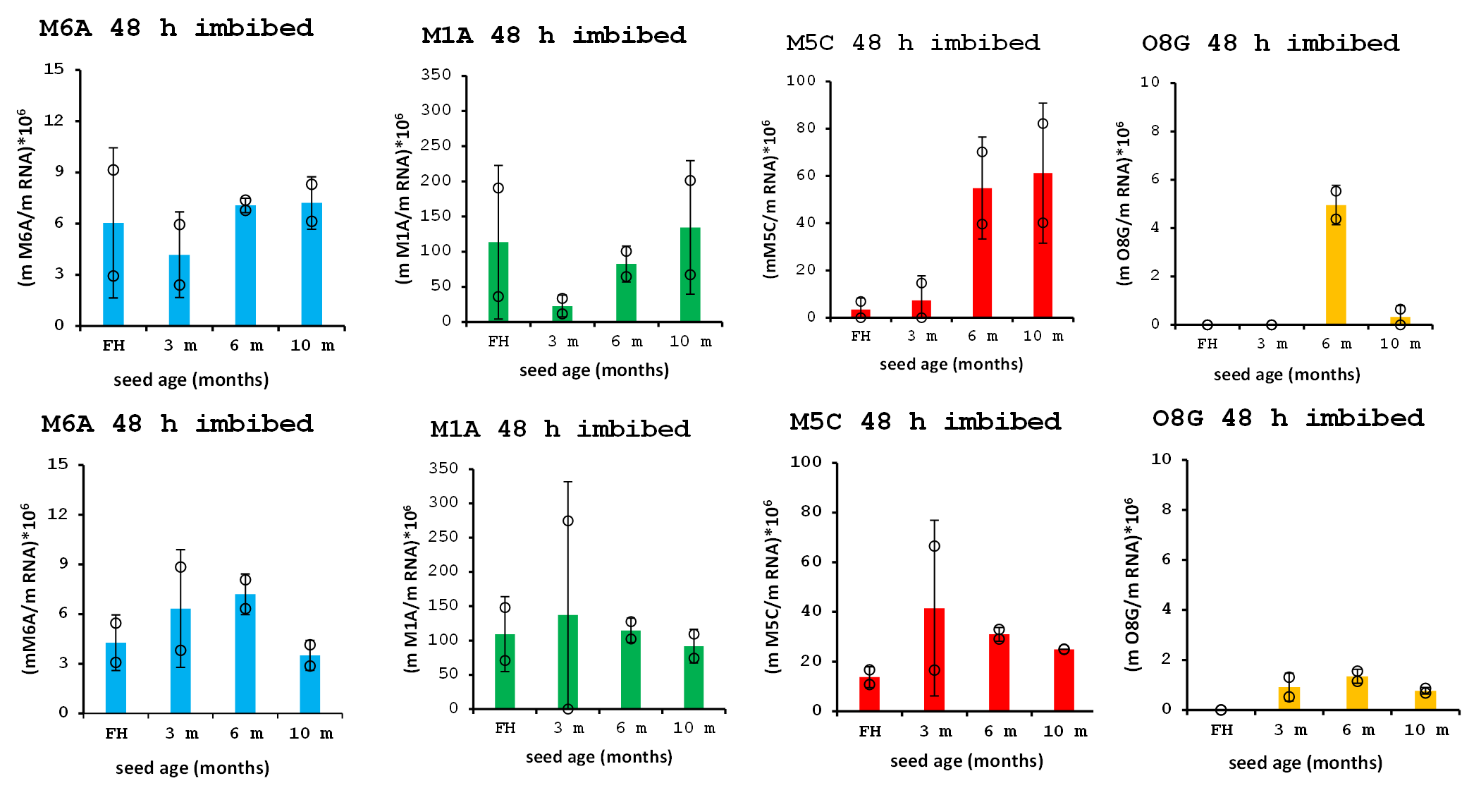


**Fig. S4.** Content of m6A (N-6-methyladenosine), m1A (N-1-methyladenosine), m5C (5-methylcytidine) and o8G (8-oxoguanosine) RNA modifications in FH (freshly harvested) and 3, 6 and 10-month after-ripened seeds of a) Col and b) Cvi after 48-h imbibition. Data are expressed as a relation to total RNA (circles show the content of modified bases in two biological replicates, each represents an average of three analytical measurements).


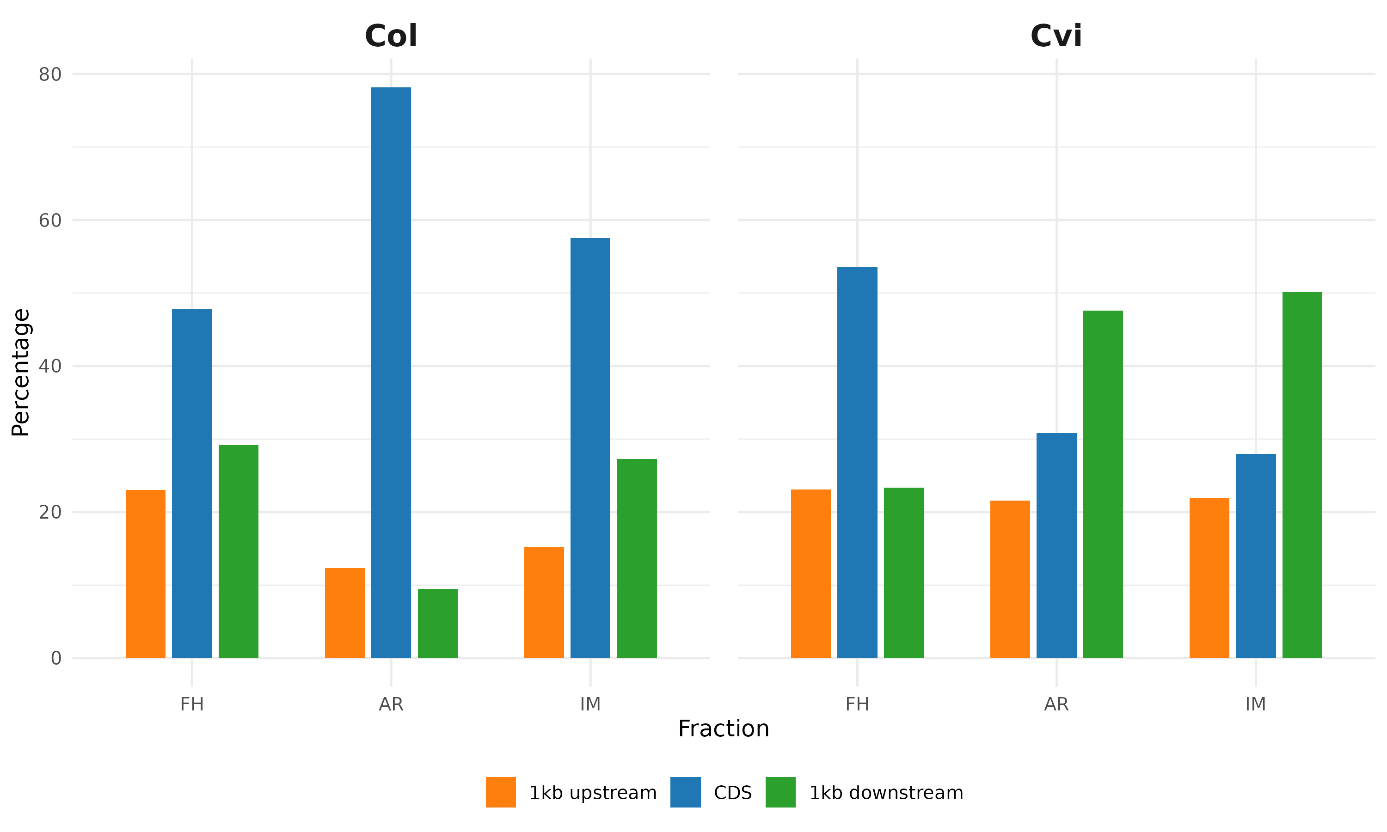


**Fig. S5.** Localization of m6A identified peaks within the 1kb upstream region, coding sequence (CDS) and 1 kb downstream region of the genes. The data show the percentual representation of peak localization. To determine the localization of m6A peaks, the peak centres were calculated in Bash from the exomePeak2 output and intersected with the corresponding Araport11 genomic features (1 kb upstream, CDS, and 1 kb downstream regions) using BEDTools. Obtained results were visualized in R (version 4.5) using the ggplot2 package.


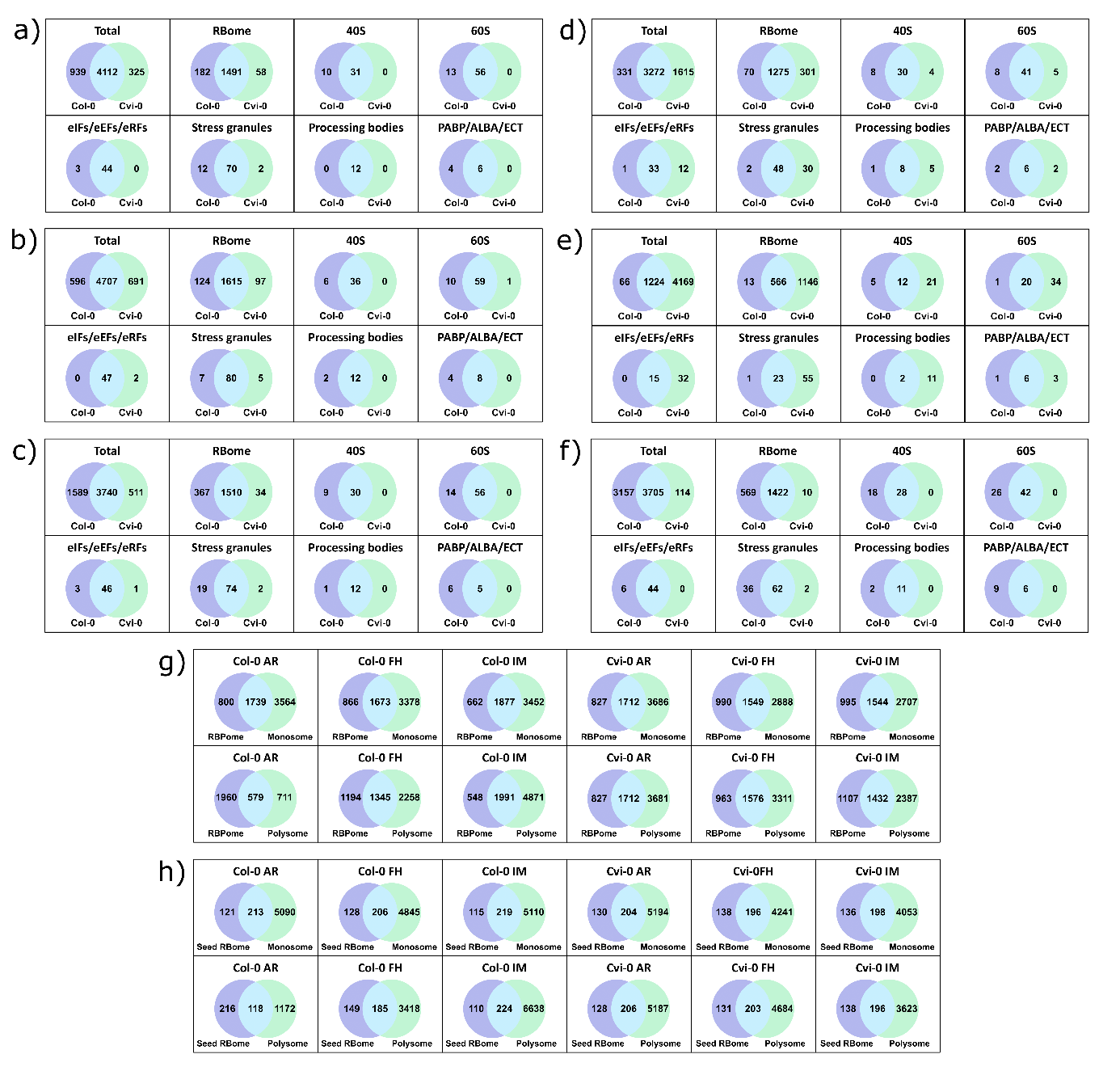


**Fig. S6:** Comparative analysis of monosomal and polysomal fractions comparing Col and Cvi. Venn diagrams show comparison of proteins present in various functional group present in monosomes of Col and Cvi at a similar stage - **a)** freshly harvested, **b)** after-ripened, **c)** imbibed seeds. Analogically, polysome fraction comparison is shown at **d)** freshly harvested, **e)** after-ripened, **f)** imbibed seeds. Functional protein groups were classified into total proteins, RNA-binding proteins (RBPome), 40S and 60S ribosomal subunits, eIFs/eEFs/eRFs, stress granules, processing bodies, and PABP/ALBA/ECT proteins. Venn diagrams of proteins present in individual fractions of Col and Cvi compared to **g**) RNA-binding proteins from the complete RBPome list in the dataset of Zhang *et al.* 2023 and **h)** seed mRNA-binding proteome reported by Sajeev *et al.*(2022). RBPome comparison includes monosome and polysome fractions at AR, FH and IM Col and Cvi seeds.

**Supplementary Files**

**Supplementary File 1.** Percentage of seed germination at 0, 24, 48 and 72h intervals for Col a Cvi seeds at different harvest times (FH, AF).

**Supplementary File 2.** TMM-normalized RNA-seq counts for all genes across all samples used for mRNA sequencing.

**Supplementary File 3.** GO terms analysis of genes of the pre-mono, mono- and polysomal fractions isolated from Col and Cvi genotypes in different stages.

**Supplementary File 4.** GO terms analysis of unique and shared genes of pre-mono- and monosomal fractions in comparison between Col and Cvi genotypes.

**Supplementary File 5.** Enrichment analysis of m^6^A-modified genes unique and shared among studied germination stages of Col and Cvi seeds.

**Supplementary File 6.** Enrichment analysis of m^6^A-modified genes unique and shared between Col and Cvi seeds.

**Supplementary File 7.** GO terms analysis of genes containing sequences with a special motif.

**Supplementary File 8.** Enrichment analysis of proteins identified in monosomal and polysomal fractions of Col and Cvi seeds.
